# Reactivity of a Ruthenium–Carbonyl Complex in the Methanol Dehydrogenation Reaction

**DOI:** 10.1002/cctc.201600709

**Published:** 2016-08-18

**Authors:** Fenna F. van de Watering, Martin Lutz, Wojciech I. Dzik, Bas de Bruin, Joost N. H. Reek

**Affiliations:** ^1^ Homogeneous, Supramolecular and Bio-Inspired CatalysisVan't Hoff Institute for Molecular SciencesUniversity of AmsterdamScience Park 9041098 XHAmsterdamThe Netherlands; ^2^ Crystal and Structural Chemistry, Bijvoet Center for Biomolecular ResearchFaculty of ScienceUtrecht UniversityPadualaan 83584 CHUtrechtThe Netherlands

**Keywords:** dehydrogenation, homogeneous catalysis, hydrogen, ruthenium, salen ligands

## Abstract

Finding new catalysts for the release of molecular hydrogen from methanol is of high relevance in the context of the development of sustainable energy carriers. Herein, we report that the ruthenium complex Ru(salbinapht)(CO)(P*i‐*Pr_3_) {salbinapht=2‐[({2′‐[(2‐hydroxybenzyl)amino]‐[1,1′‐binaphthalen]‐2‐yl}imino)methyl]phenolato} (**2**) catalyzes the methanol dehydrogenation reaction in the presence of base and water to yield H_2_, formate, and carbonate. Dihydrogen is the only gas detected and a turnover frequency up to 55 h^−1^ at 82 °C is reached. Complex **2** bears a carbonyl ligand that is derived from methanol, as is demonstrated by labeling experiments. The carbonyl ligand can be treated with base to form formate (HCOO^−^) and hydrogen. The nature of the active species is further shown not to contain a CO ligand but likely still possesses a salen‐derived ligand. During catalysis, formation of Ru(CO)_2_(H)_2_(P‐*i*Pr_3_)_2_ is occasionally observed, which is also an active methanol dehydrogenation catalyst.

The transformation to a society based on renewable energy requires both the harvesting of sustainable energy (wind and solar) as well as the transformation of this energy into proper energy carriers. In this context, the use of H_2_ is an attractive energy carrier, as it can be generated with sustainable energy by water splitting, and its energy can be released by combustion or in a fuel cell to provide water as the only byproduct.^1^, ^2^, ^3^, ^4^ The storage of molecular hydrogen as a gas is challenging, as it has low volumetric density.^5^ One possibility to overcome this limitation is to store H_2_ reversibly in the form of a different chemical energy carrier. In this context, methanol is an attractive hydrogen carrier, as it is a liquid at room temperature and contains a substantial amount (12.6 %) of hydrogen.^6^ The use of methanol as a hydrogen‐storage requires the development of catalysts that allow reversible dehydrogenation of methanol to CO_2_ under mild reaction conditions. Dehydrogenation of methanol to carbon dioxide typically proceeds by the three steps depicted in Scheme 1: The release of the first equivalent of H_2_ results in the formation of formaldehyde, which upon reaction with 1 equivalent of water releases the second equivalent of H_2_ to yield formic acid. Finally, formic acid is dehydrogenated to form carbon dioxide, which produces the third equivalent of H_2_. Under basic conditions, carbon dioxide is trapped in the form of carbonate, which leads to a clean gas outlet of pure hydrogen.

**Scheme 1:**
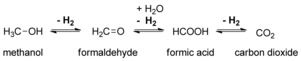
Proposed intermediates in the transition‐metal‐catalyzed methanol dehydrogenation reaction.

The full dehydrogenation of methanol to CO_2_ in the presence of water was only reported in 2013 by the groups of Beller^7^ and Grützmacher.^8^ Their systems consisted of ruthenium catalysts capable of performing this reaction in aqueous media at temperatures below 100 °C. In the absence of base, Beller's ruthenium–PNP (Ru–PNP=RuHCl(CO)(PNP), PNP=HN(C_2_H_4_P*i*‐Pr_2_)_2_) system reached a turnover frequency (TOF) of almost 22 h^−1^ and Grützmacher's ruthenium–trop_2_dad (Ru–trop_2_dad=K(dme)_2_][RuH(trop_2_dad), trop_2_dad = 1,4‐*bis*(5H‐dibenzo[a,d]cyclohepten‐5‐yl)‐1,4‐diazabuta‐1,3‐diene) system reached a TOF of 54 h^−1^. The addition of KOH led to an almost 30‐fold increase in activity (TOF=613 h^−1^) for Beller's system.^9^ Dehydrogenation of paraformaldehyde in the presence of water, which releases 2 equivalents of hydrogen, was also reported by the group of Prechtl.^10^

Previously, our group reported new catalysts for the (reversible) dehydrogenation of formic acid.^11^, ^12^, ^13^, ^14^ In search of new catalytic systems for the full dehydrogenation of methanol, we turned our attention to salen‐type ligands. These ligands can be easily prepared by a one‐step condensation, which in principle gives facile access to high structural diversity. An active system could thus easily be tuned by changing, for example, the steric and electronic properties of the ligand.^15^, ^16^, ^17^ Notably, complexes of ruthenium and iron with Schiff‐base‐derived ligands were previously shown to activate alcohols in catalytic transfer‐hydrogenation reactions.^18^, ^19^, ^20^, ^21^, ^22^

We aimed at the synthesis of a ruthenium complex with the “salbinapht” ligand [salbinapht=(*R*)‐2′2′‐bis(salicylideneamino)‐1,1′‐binaphtyl, **1**]. This ligand features a binaphthyl backbone that enforces a *cis*‐β geometry around the metal. Such geometry enables two *cis* vacant sites in the metal coordination sphere,^23^, ^24^ which could facilitate an inner‐sphere dehydrogenation process. Our initial attempts to synthesize a ruthenium complex with the salbinapht ligand revealed that upon treating [RuCl_2_(dmso)_4_] with **1** in methanol in the presence of LiOMe and triisopropylphosphine, complex **2** was formed in a moderate yield of approximately 20 % (Scheme 2).

**Scheme 2:**
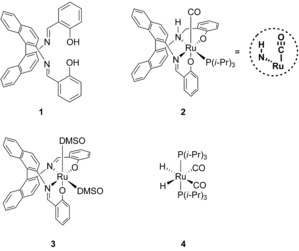
Compounds discussed in this article. Complex **4** was previously reported by Werner et al.^26^

The ^1^H NMR and ^31^P NMR spectra of **2** confirm the presence of one triisopropylphosphine ligand coordinated to the ruthenium center. The aromatic region in the ^1^H NMR spectrum of **2** shows the loss of symmetry of the salbinapht ligand, which indicates that the *cis*‐β geometry is indeed adopted.^25^ Interestingly, only one imino proton (*δ*=7.1 ppm) can be identified in the ^1^H NMR spectra (see Figures S4–S8 in the Supporting Information) and new signals appear in the region between *δ*=3.5 and 5.5 ppm that correspond to a −CH_2_NH− moiety. This suggests that one of the imine groups of ligand **1** is hydrogenated during complex formation. IR spectroscopy reveals an intensive band at *ν*=1919 cm^−1^ indicative of the presence of a CO ligand. This carbonyl ligand must stem from methanol/methoxide used in the synthesis of the complex, as no other CO source was used. Thus, the spectroscopic analysis suggests that complex **2** is a six‐coordinate species containing one tetradentate dihydrosalbinapht, one triisopropylphosphine, and one carbonyl ligand.

Recrystallization of complex **2** from acetonitrile gave yellow‐orange crystals suitable for single‐crystal X‐ray diffraction. The crystal structure (Figure 1) is in accordance with the spectroscopic analyses and reveals the expected octahedral coordination environment around ruthenium. Ligand **1**‐H_2_ is coordinated in a *cis‐*β fashion with one of the π‐donating phenolato groups coordinated *trans* to the π‐accepting carbonyl and the other *trans* to the imine. As a consequence, the σ‐donating phosphine is coordinated *trans* to the σ‐donating amine. To date, only two examples of the regioselective monoreduction of a chiral salen ligand have been reported.^27^, ^28^ Complex **2** has three stereocenters (i.e., Ru, N, and binaphthyl backbone). Remarkably, only one diastereoisomer of **2** is formed during the synthesis. The enantiopurity of the crystal structure in the noncentrosymmetric space group *P*1 was confirmed by using the Flack parameter (see the Supporting Information, crystal data).

**Figure 1:**
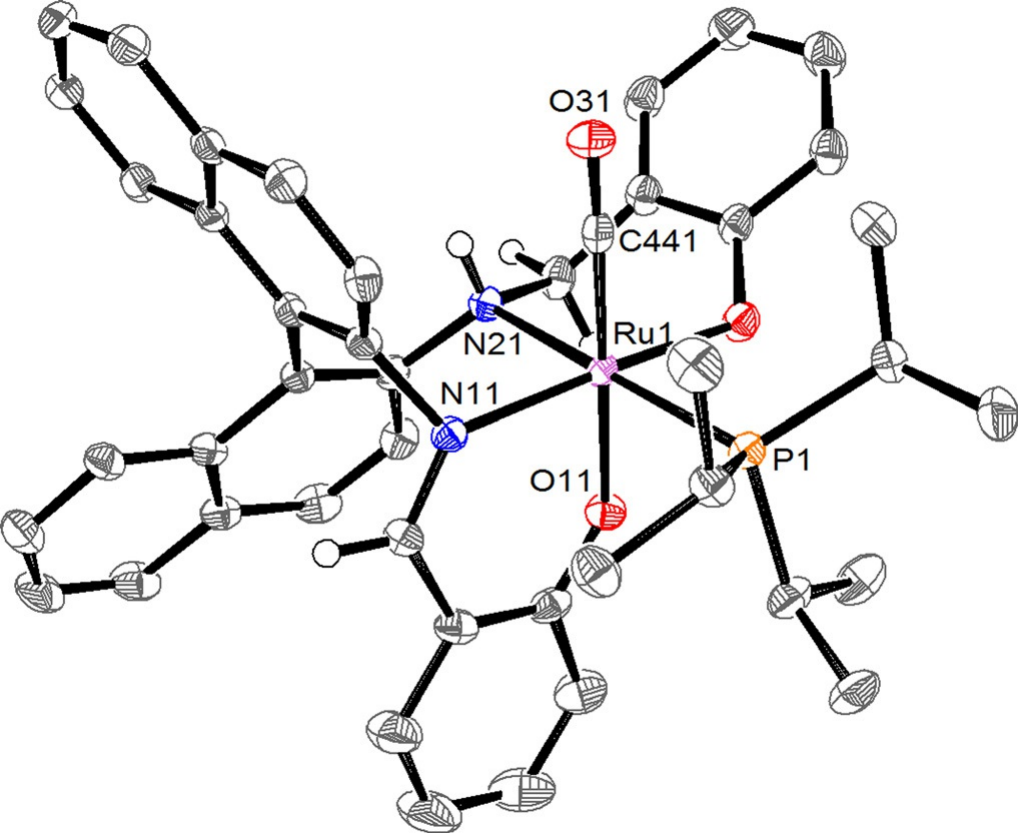
ORTEP drawing of **2** (50 % probability ellipsoids).^29^ Only one of two independent molecules is shown. Acetonitrile molecules and most hydrogen atoms are omitted for clarity.

Given that complex **2** is coordinatively saturated, it was not clear that it would catalyze the dehydrogenation of methanol. However, we anticipated that under basic conditions the carbonyl ligand could undergo the water‐gas‐shift reaction with water present in the reaction mixture.^30^, ^31^, ^32^ Therefore, we investigated the activity of **2** as a catalyst in the methanol dehydrogenation reaction (Table 1). Dioxane was used as a cosolvent during the catalytic reaction, as complex **2** has low solubility in methanol / water. Hydrogen evolution was measured volumetrically. Samples of the evolved gas were analyzed by using gas chromatography. Molecular hydrogen was always the only gas formed and no CO was detected.

**Table 1:** Hydrogen generation from methanol with complex **2**.^[a]^

Entry	Base	Concentration of base [m]	Additional solvent	*T*_in_^[b]^ [°C]	TOF [h^−1^]
1	KOH	8	water	82	55
2	KOH	6	water	79	37
3	KOH	4	water	76	29

[a] Reaction conditions: 30 mL of solvent (25 % dioxane / 75 % methanol: water (9:1 v/v), catalyst (≈12 μmol), base as described, reaction time of 4.5 h, all experiments were measured in duplicate. The oil bath was set to 110 °C to ensure reflux conditions for all measurements. [b] Internal temperature of the refluxing solution.

In the 8 m KOH solution experiment, complex **2** showed moderate activity (TOF=55 h^−1^; Table 1, entry 1). A decrease in the amount of base led to lower reaction rates, which can (partially) be explained by the effect of the lower internal reaction temperature (resulting from a lower boiling point of the reaction mixture; Table 1, entries 2 and 3). Analysis of the reaction mixture revealed the presence of both formate (HCOO^−^) and carbonate (CO_3_^2−^) salts (Table 1, entry 1 after extraction with CH_2_Cl_2_ and by using NMR spectroscopy. See Figures S20 and S21). The presence of these salts shows that water is involved in the overall dehydrogenation reaction, yielding up to 3 equivalents of hydrogen.

Next, we investigated the mechanism of the methanol dehydrogenation reaction by our system. We first studied the sources of both the protons on the hydrogenated imine and the carbonyl ligand. To confirm the origin of the protons and the carbonyl ligand, we conducted isotope‐labeling experiments. The labeled complexes of **2** were synthesized by using either CD_3_OD or ^13^CH_3_OH as the solvent. The ^1^H NMR spectrum of complex **2**‐D_2_, synthesized from CD_3_OD, revealed the absence of the signals at *δ*=4.60 and 5.34 ppm owing to the presence of deuterons at the reduced imine position. The use of ^13^CH_3_OH for the synthesis of **2**‐^13^CO confirmed that the carbonyl ligand is derived from methanol: the carbonyl signal in the ^13^C NMR spectrum [*δ*=205.30 ppm, d, ^2^ *J*(^31^P,^13^C)=16.9 Hz] was the only signal found in this region, and the ^31^P NMR spectrum revealed a doublet at *δ*=60.43 ppm that is a result of coupling with ^13^CO [^2^ *J*(^31^P,^13^C)=17.7 Hz]. These labeling experiments confirmed that methanol is the source of both the amine protons and the carbonyl ligand in complex **2**.

The formation of ruthenium hydrido carbonyl complexes derived from alcohols in the presence of phosphine ligands is a well‐established reaction,^9^, ^26^, ^33^, ^34^ and therefore, it seemed likely that the dehydrogenation of methanol occurred before coordination of **1**. Therefore, we also performed the synthesis of **2** in two steps: first preformation of a putative Ru(CO)_2_(H)_2_(P*i*Pr_3_)_2_ (**4**) species (Scheme 2),^26^ and subsequently, the addition of salbinapht ligand **1**. This modified procedure yielded **2** in much higher yield (80 %), which suggests a mechanism in which in situ formed species **4** reacts with salbinapht ligand **1** to form **2**. Thus, the methanol dehydrogenation reaction occurs first in the presence of ruthenium–phosphine species, and this is followed by the reduction of one of the imine groups of ligand **1**, which leads to complex **2** as a single diastereoisomer.

Subsequently, we investigated whether the carbonyl ligand was an intermediate in the methanol dehydrogenation reaction. The first experiment was conducted with regard to the extrusion of the CO ligand from **2** to create a vacant site for methanol coordination. We anticipated that the water‐gas‐shift reaction may play a role in this process and, therefore, studied the effect of the base present in the reaction mixture. An NMR spectroscopy experiment was conducted, in which ^13^CO‐labeled complex **2** was treated with base in a high‐pressure NMR tube at elevated temperatures (Scheme 3, experiment 1). For this experiment, we used the same reactant ratios as those used during the catalysis experiment in Table 1 (entry 1). Heating **2**‐^13^CO in a KOH solution of MeOH, H_2_O, and THF at 100 °C resulted in the total disappearance of the ruthenium carbonyl signal within 3.5 h (see Figure S22). Dissolution of the white precipitate that had formed during the reaction in D_2_O showed the presence of ^13^C‐enriched potassium formate (as evidenced by ^1^H NMR and ^13^C NMR spectroscopy). Analysis of the headspace showed the presence of molecular hydrogen (as evidenced by gas chromatography). From this data, we conclude that the CO ligand that was obtained from methanol (as evidenced earlier by the formation of **2**‐^13^CO from ^13^CH_3_OH), can be further treated with base to result in the formation of potassium formate.

**Scheme 3:**
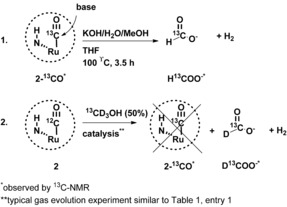
Investigation of the role of the CO ligand. Experiment 1 shows that the ^13^CO ligand can be extruded with base to form the ^13^C‐enriched formate ion (H^13^COO^−^). Experiment 2 shows that the ^13^C‐enriched CO ligand is not regenerated during catalysis if ^13^CD_3_OD is used as the substrate for catalysis. The formation of the ^13^C‐enriched formate ion (H^13^COO^−^) indicates that ^13^CD_3_OD is dehydrogenated by ruthenium. Consequently, complex **2** is probably a precatalyst and the active species does not bear a carbonyl ligand.

Importantly, several current homogenous methanol dehydrogenation catalysts feature “spectator” CO ligands.^7^, ^35^, ^36^, ^37^, ^38^ In view of the above results, it is plausible that under the catalytic conditions these catalysts can lose CO through nucleophilic attack of base present in the reaction mixture to create a new vacant site. This extra vacant site could also play a role in the overall mechanism for these catalysts.

Subsequently, we investigated if complex **2** was regenerated from methanol during catalysis after its attack by base. Therefore, we applied standard reaction conditions similar to those shown in Table 1, entry 1 (8 m KOH, 25 % dioxane / 75 % methanol:water (9:1 v/v), 12 μmol of catalyst at reflux temperature) but this time in the presence of 50 % labeled ^13^CD_3_OD (Scheme 3, experiment 2). After 2 h, the still‐active reaction mixture was extracted with dichloromethane, and its ^13^C NMR spectrum was measured. No ^13^C‐enriched ruthenium–carbonyl signal corresponding to **2**‐^13^CO was detected. However, ^13^C NMR and ^1^H NMR spectra of an aliquot of the water phase in D_2_O did show the presence of H/D^13^COO^−^ (see Figures S23 and S24). The presence of formate indicates that the ^13^C‐labeled methanol is dehydrogenated over the course of 2 h and that the resting state of the actual catalyst is not coordinated with a carbonyl ligand. ^1^H NMR spectroscopy revealed the presence of a salen‐derived species that had a ^1^H NMR spectrum different from that of ligand **1** and that of complex **2**. Unfortunately, we were unable to determine the complete structure of this salen species, as it was not possible to isolate the compound in pure form from the complex reaction mixture.

The above‐described experiments show that the carbonyl ligand of complex **2** can be extruded under the basic conditions used during catalysis. The formation of the labeled catalyst (i.e., **2**‐^13^CO) and subsequent isolation of the labeled formate (i.e., H^13^COO^−^) after the reaction with base show the first two steps of the methanol dehydrogenation with ruthenium. The fact that labeled complex **2**‐^13^CO was not regenerated from ^13^CH_3_OH during catalysis shows that complex **2** is not the resting state during catalysis. Probably other unidentified salen‐derived species that lack a CO ligand are formed, but these could not be identified.

Trace amounts of complex **4** were occasionally found after the catalytic reactions and (as complex **2** is prepared from complex **4**) its performance in the dehydrogenation reaction was investigated. The activity of complex **4** was studied under conditions similar to the experiment described in Table 1, entry 1 (see Figure S19) and activity (TOF=50 h^−1^) similar to that of complex **2** was revealed. Thus, it is possible that in situ formed **4** is at least partially responsible for methanol dehydrogenation during catalysis.

The above result leaves the question as to whether the active species generated from **2** is still coordinated to a salbinapht ligand. Therefore, we investigated the activity of [Ru(**1**)(dmso)_2_] (**3**) (an analogue of **2**, see Scheme 2) that does not feature a phosphine or a carbonyl ligand in the methanol dehydrogenation reaction. Upon subjecting complex **3** to the catalytic conditions (similar to Table 1, entry 1), it showed hydrogen evolution in the first 30 min (Turnover number, TON_30 min_=23), as determined volumetrically and as analyzed by gas GC (see Figure S19). This suggests that a ruthenium center supported with a salbinapht ligand (**1**) is capable of catalyzing the dehydrogenation of methanol. However, under the applied conditions the catalyst has low stability, and the presence of a coordinated phosphine clearly has a beneficial influence on the overall performance of complex **2**.

In conclusion, we demonstrated that the ruthenium complex based on salen ligand **1** provides an active catalyst for the dehydrogenation of methanol, in which molecular hydrogen, formate, and carbonate are formed. Mechanistic investigations demonstrated that the CO ligand on the carbonyl ruthenium complex can be attacked by a nucleophile (KOH or H_2_O) to form potassium formate. The carbonyl complex is, however, not the resting state of the reaction. Future experiments should focus on the design of more active and stable catalysts systems; this may be achieved by moving to complexes based on phosphino‐Schiff bases, as they are already used for transfer‐hydrogenation reactions. Initial experiments with such ruthenium complexes show that they can indeed dehydrogenate methanol into molecular hydrogen.

## Supporting information

As a service to our authors and readers, this journal provides supporting information supplied by the authors. Such materials are peer reviewed and may be re‐organized for online delivery, but are not copy‐edited or typeset. Technical support issues arising from supporting information (other than missing files) should be addressed to the authors.

Supplementary
