# Supplementary material for: Reactivity of a Ruthenium–Carbonyl Complex in the Methanol Dehydrogenation Reaction
Source: ChemCatChem. 2016 Aug 18;8(17):2752–6. doi: 10.1002/cctc.201600709 (PMC5129501; doi:10.1002/cctc.201600709)
Supplement: Supplementary file 1 — Supplementary [file CCTC-8-2752-s001.pdf]

Heterogeneous & Homogeneous & Bio- & Nano-
CHEMCATCHEM
CATALYSIS

Supporting Information

Reactivity of a Ruthenium–Carbonyl Complex in the Methanol Dehydrogenation Reaction

Fenna F. van de Watering,[a] Martin Lutz,[b] Wojciech I. Dzik,[a] Bas de Bruin,[a] and Joost N. H. Reek*[a]

cctc_201600709_sm_miscellaneous_information.pdf

Experimental Section

Table of Contents

1) General methods 1

2) Synthesis of new compounds 1

3) Hydrogen evolution experiments 11

4) Mechanistic investigations 15

5) Crystal data for **2** 18

1) General methods

All reactions were carried out under an inert (argon/nitrogen) atmosphere using standard Schlenk techniques. THF was distilled from sodium benzophenone ketyl; acetonitrile, methanol and dichloromethane were distilled from CaH_2 and dioxane was distilled from sodium all under nitrogen. NMR spectra (^{1}H, ^{13}C and ^{31}P, H-H COSY and HSQC) were measured on a Bruker AMX 400, a Varian Mercury 300, a Bruker DRX 500, or a Bruker DRX 300 spectrometer. Infrared spectra were recorded on a Thermo Nicolet NEXUS 670 FT-IR. The high resolution mass spectrum were recorded on a JEOL AccuTOF LC, JMS-T100LP mass spectrometer using electron spray ionization (ESI) on a JEOL AccuTOF GC v 4g, JMS-T100GCV mass spectrometer using field desorption (FD).

2) Synthesis of new compounds

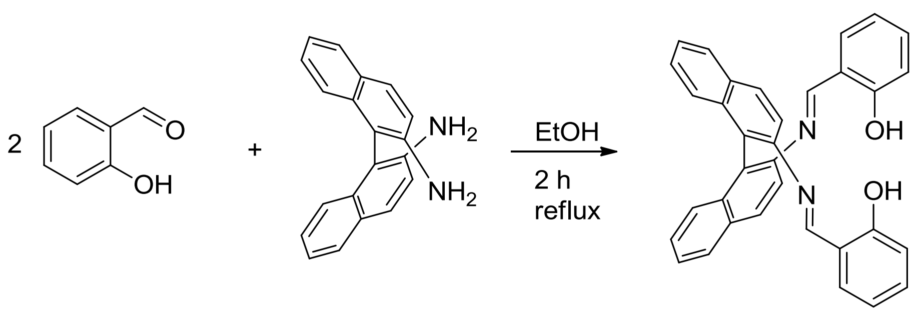

1

Figure 1: Synthesis of (R)-2,2'-bis(salicylideneamino)-1,1'-binaphthyl (**1**)

(R)-2,2'-bis(salicylideneamino)-1,1'-binaphthyl (1): a solution of 1.05 g of (R)-1,1-binapthyl-2,2’-diamine (3.67 mmol) in ethanol (25 mL) was transferred to a 100 mL flame dried schlenk flask equipped with a reflux condenser. To this chalky brown suspension 0.80 mL of the salicylaldehyde (7.51 mmol) dissolved in 30 mL ethanol was added drop wise and heated under reflux for 2h. The reaction mixture was cooled to room temperature and concentrated to 4-5 mL. The precipitated yellow product was then filtered off and washed with ethanol (1 x 5 mL) and pentane (2 x 5 mL) and dried to give ligand **1** in pure form (1.71 g, 94 %). Spectra were in agreement with literature.[1,2]

^{1}H NMR (500 MHz, DMSO-d_6): δ 12.24 (s, 2H), 9.08 (s, 2H), 8.32 – 8.18 (m, 2H), 8.08 (d, J = 8.1 Hz, 2H), 8.00 (d, J = 9.0 Hz, 2H), 7.48 (d, J = 7.9 Hz, 4H), 7.31 (s, 2H), 7.23 (s, 2H), 7.05 (d, J = 8.5 Hz, 2H), 6.83 (s, 2H), 6.61 (d, J = 8.2 Hz, 2H). **^{13}C** NMR (126 MHz, DMSO d_6): δ 162.73, 160.03, 142.90, 133.10, 132.71, 132.60, 132.25, 129.89, 129.56, 128.38, 127.11, 125.97, 125.67, 119.08, 118.87, 117.15, 116.40, 40.11, 40.02, 39.94, 39.86, 39.78, 39.69, 39.61, 39.52, 39.44, 39.35, 39.19, 39.02.

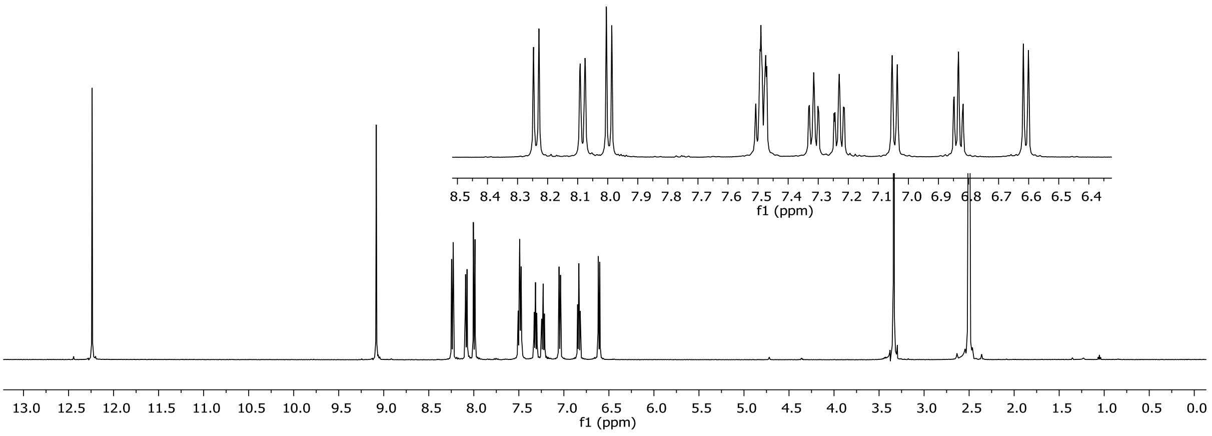

Figure S2: ^{1}H-NMR spectrum (DMSO-d_6) of ligand **1**

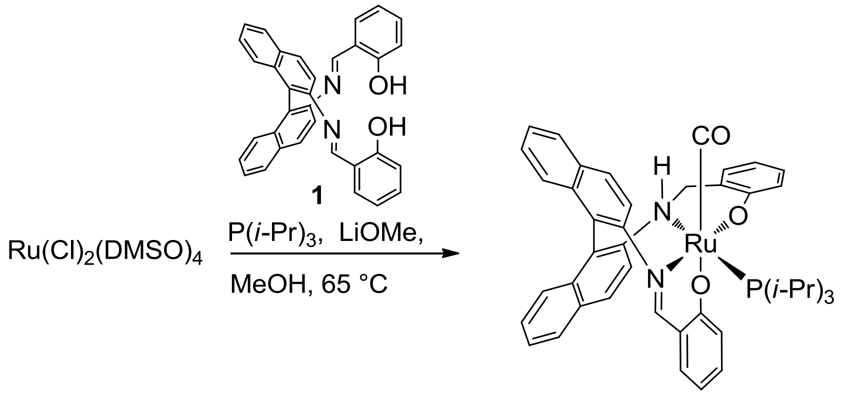

2

Figure S3: Synthesis of Ru(1-H_2)(CO)P(i-Pr_3)·MeCN (**2**)

Ru(1-H_2)(CO)P(i-Pr_3)·MeCN (2): 100 mg (0.21 mmol) of $Ru(Cl)_2(DMSO)_4$ was transferred to a flame dried schlenk flask equipped with a reflux condenser and the vessel was purged three times with argon-vacuum cycles. Then 10 mL of methanol was added, followed by the addition of 80 μL (0.42 mmol) of P(i-Pr)$_3$. The colourless solution with suspended yellow solid was heated under reflux until the solid had dissolved and the solution turned orange-red (approximately 5 minutes). The reaction mixture was cooled to room temperature after which 0.42 mL of a LiOMe solution (0.42 mmol, 1M in methanol) was added and stirred for 5 minutes. Then the orange solution was brought to reflux for 30 minutes. In the meantime 102 mg (0.21 mmol) of (R)-2,2'-bis(salicylideneamino)-1,1'-binaphthyl (**1**) was transferred to a different flame dried Schlenk flask, which was purged three times with argon-vacuum cycles. To this yellow solid 20 mL of methanol and 0.42 mL (2.55 mmol) of LiOMe-solution (1M in methanol) where added. The yellow suspension was added via a syringe to the stirring solution containing the ruthenium phosphine complex. The reaction mixture was heated for

45 min which resulted in an orange solution. The reaction mixture was concentrated in vacuo to approximately 2 mL, leading to the formation of an orange-yellow precipitate. The filtrate was removed using a syringe and the orange-yellow solid was dried and subsequently recrystallized from acetonitrile giving the orange-yellow crystalline complex in pure form. The acetonitrile filtrate was concentrated to ~1 mL giving a second crop of **2**. Overall yield: 148 mg, 90 %.

Only one diastereoisomer is formed during the synthesis: the N-atom of the amine group (Figure S3, N-H) has S configuration, the ruthenium centre is coordinated in a OC-6-56-A configuration[3], and binapthyl remains in R configuration.

^{1}H NMR (500 MHz, MeCN-d_3): δ 8.12 (d, *J* = 8.7 Hz, 1H), 8.02 (d, *J* = 8.3 Hz, 1H), 7.70 (d, *J* = 8.3 Hz, 1H), 7.68 – 7.62 (m, 2H), 7.60 (d, *J* = 8.7 Hz, 1H), 7.51 (t, *J* = 7.6 Hz, 1H), 7.37 (t, *J* = 7.7 Hz, 1H), 7.33 (t, *J* = 7.4 Hz, 1H), 7.22 (d, *J* = 8.6 Hz, 1H), 7.16 (t, *J* = 7.8 Hz, 1H), 7.10 (s, 1H), 7.05 (t, *J* = 8.7, 6.9, 1.8 Hz, 1H), 7.00 (d, *J* = 7.2 Hz, 1H), 6.95 (t, *J* = 7.6 Hz, 1H), 6.76 (d, *J* = 8.6 Hz, 1H), 6.66 (d, *J* = 8.6 Hz, 1H), 6.55 (d, *J* = 8.1 Hz, 1H), 6.39 (t, *J* = 7.3 Hz, 1H), 6.20 (d, *J* = 7.7 Hz, 1H), 6.06 (t, *J* = 7.3 Hz, 1H), 5.47 – 5.23 (m, 1H), 4.60 (t, *J* = 10.5 Hz, 1H), 3.76 – 3.62 (m, 1H), 2.73 – 2.46 (m, 3H), 1.26 (ddd, *J* = 123.5, 13.2, 7.2 Hz, 18H).**^{31}P** NMR (202 MHz, MeCN-d_3): δ = 60.44. **^{13}C** NMR (126 MHz, DMSO-d_6 measured in DMSO because of low solubility of the complex in MeCN): δ 204.21, 162.73, 160.03, 142.90, 133.10, 132.71, 132.60, 132.25, 129.89, 129.56, 128.38, 127.11, 125.97, 125.67, 119.08, 118.87, 117.15, 116.40, 40.11, 40.02, 39.94, 39.86, 39.78, 39.69, 39.61, 39.52, 39.44, 39.35, 39.19, 39.02. ν_{CO}= 1919 cm^{-1} Mass Analysis ESI+ [**2**H^+]: 783.2360

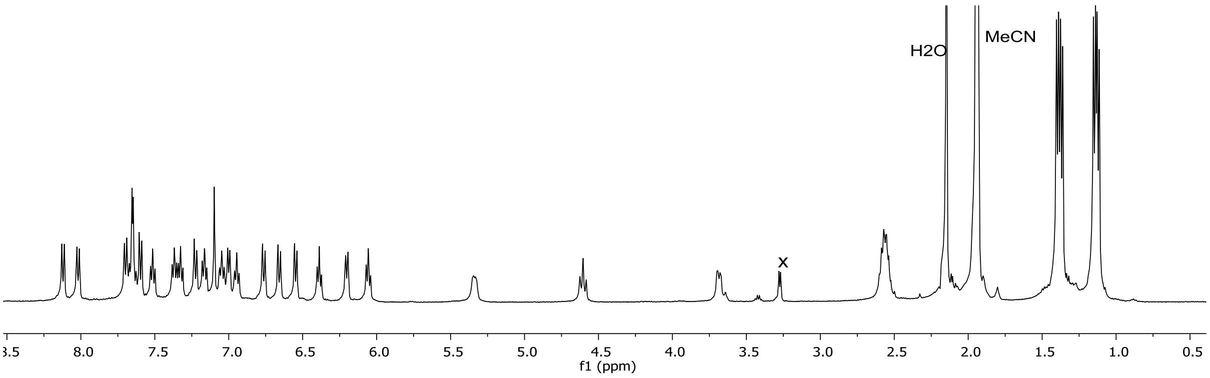

Figure S4: ^{1}H-NMR spectrum (MeCN-d_3) of **2**

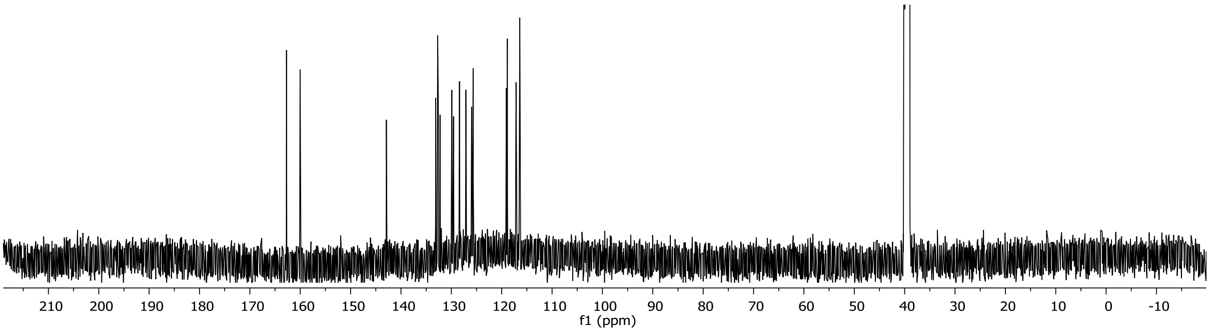

Figure S5: ^{13}C-NMR spectrum (DMSO-d_6) of **2**

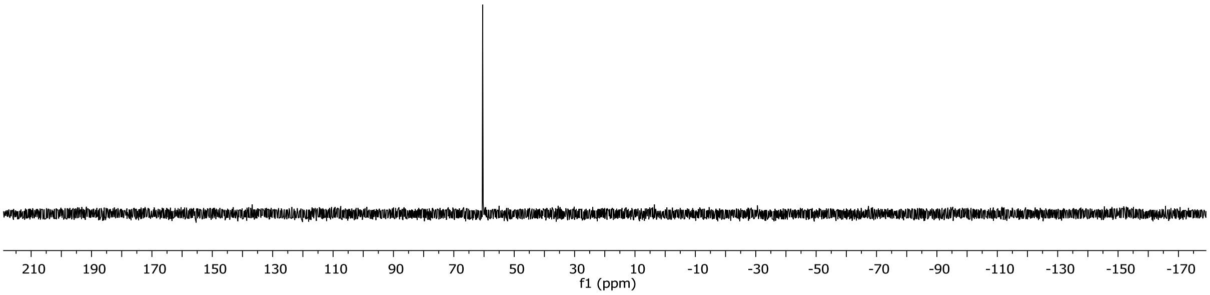

Figure S6: ^{31}P-NMR spectrum (MeCN-d_3) of **2**

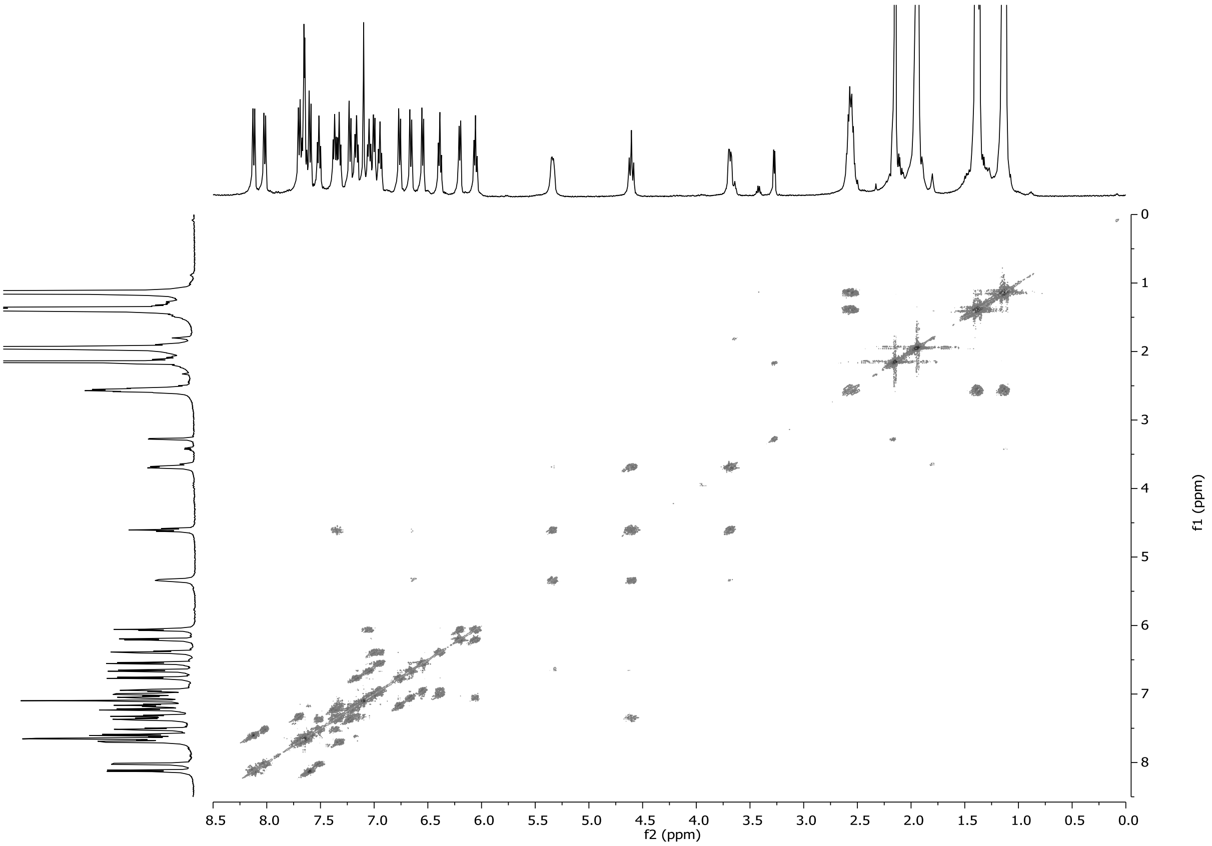

Figure S7: H-H COSY spectrum (MeCN-d_3) of **2**

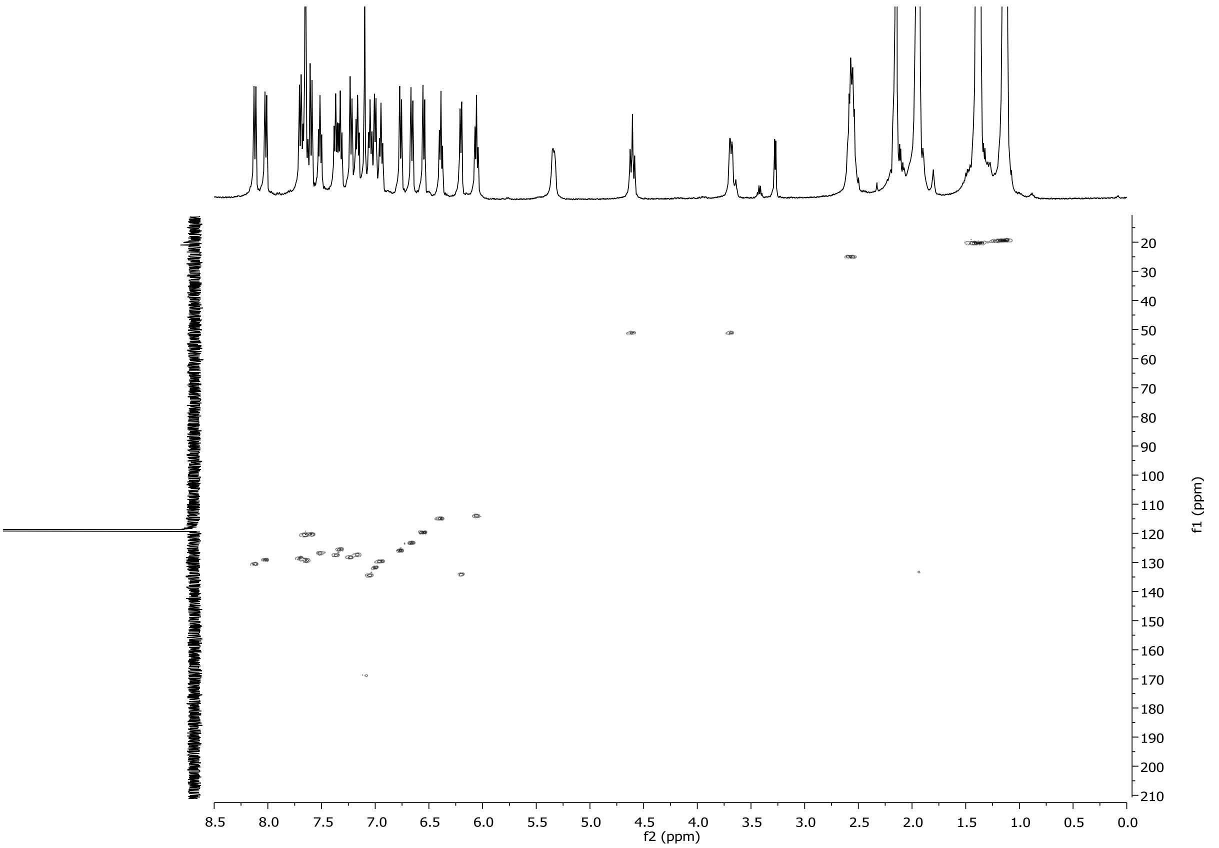

Figure S8: HSQC spectrum (MeCN-d_3) of **2**

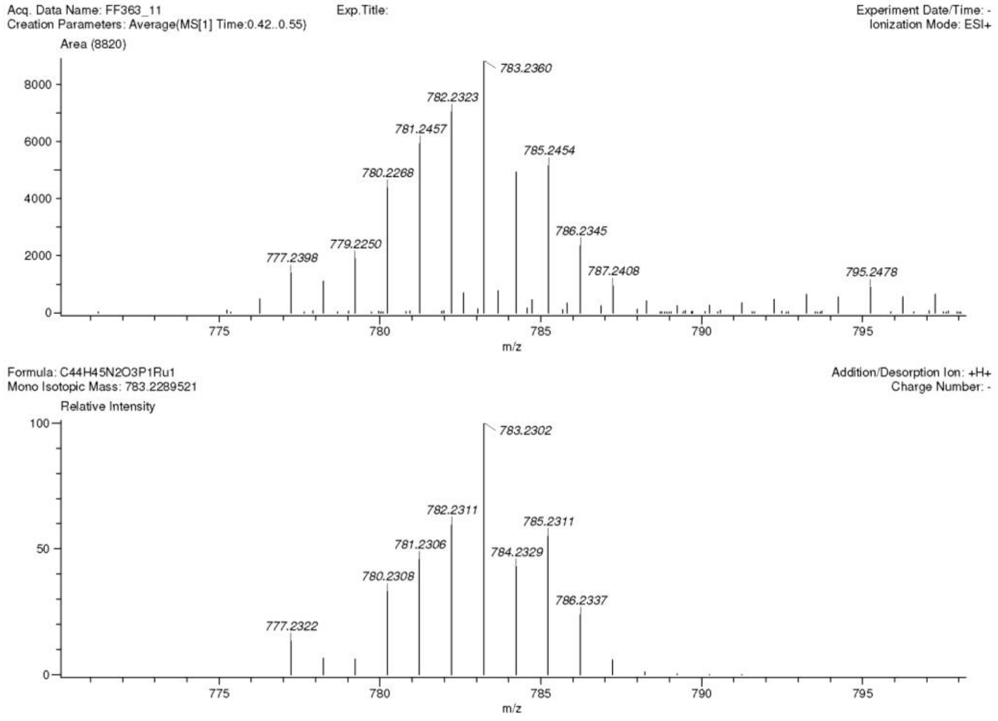

Figure S9: High resolution ESI+ mass spectrum of **2** experimental (top); simulated (bottom)

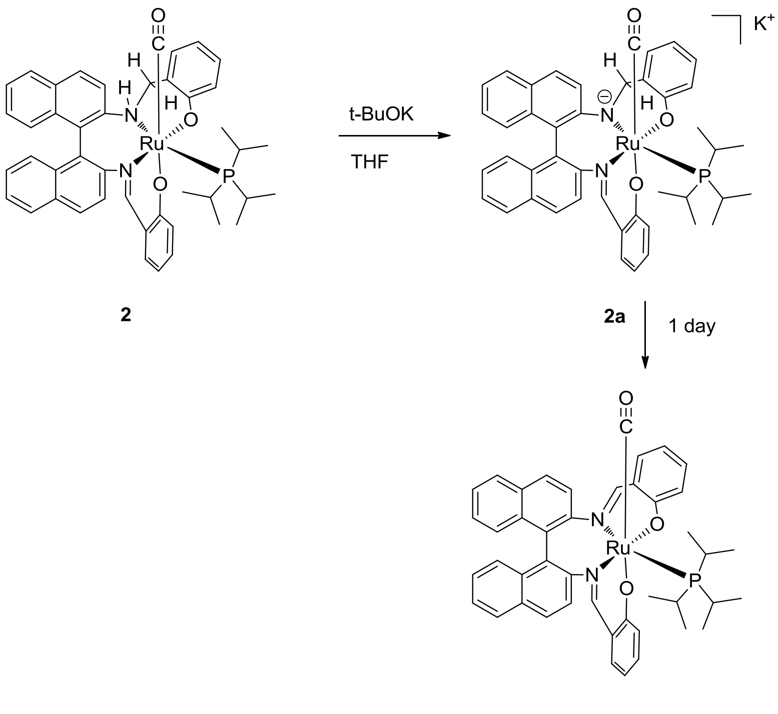

Scheme S9-a: Deprotonation of 2 forming complex 3 and 5

[Ru(1-H)(CO)P(*i*-Pr$_3$)]K (2a) ·1.5THF: 18.3 mg (0.023 mmol) of **2** was added to a flame dried schlenk and purged 3x with vacuum argon cycles. 1 mL of THF followed by 0.023 mL (0.023 mmol) of ^{t}BuOK (1M in THF) was added resulting in an immediate colour change from orange to deep red. After stirring for 5 minutes the solvent was evaporated. Although the sample was dried for 3 days under vacuum, THF present in the sample. The THF peak partly overlaps with one of the CH_2 protons. In solution, **2a** spontaneously oxidises to the double imine **2aa** within one day, which precluded the measurement of ^{13}C-NMR.

^{1}H NMR (500 MHz, DMSO-*d*$_6$): δ 7.85 (t, *J* = 9.0 Hz, 2H), 7.47 – 7.21 (m, 6H), 7.13 (t, *J* = 7.7 Hz, 1H), 6.98 (d, *J* = 8.5 Hz, 1H), 6.83 (t, 2H), 6.74 – 6.64 (m, 3H), 6.44 (d, *J* = 7.8 Hz, 1H), 6.37 (d, *J* = 8.6 Hz, 1H), 6.30 (d, *J* = 7.9 Hz, 1H), 6.26 – 6.04 (m, 2H), 5.93 (t, *J* = 7.2 Hz, 1H), 4.17 (d, *J* = 11.1 Hz, 1H), 3.58 (d, 1H), 2.58 – 2.44 (m, 3H), 1.23 (ddd, 18H). **^{31}P** NMR (202 MHz, DMSO-*d*$_6$): δ 47.93 (s).

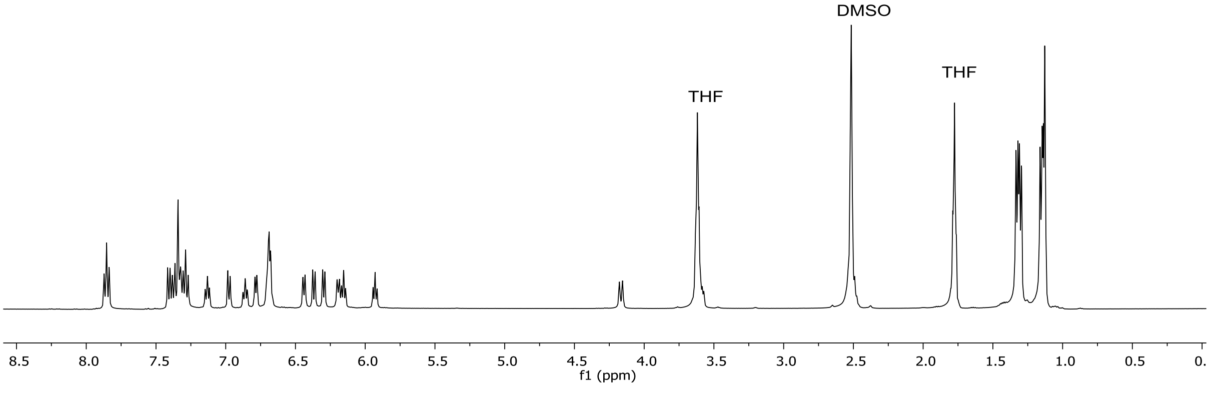

Figure S9-b: ^{1}H-NMR spectrum (DMSO-*d*$_6$) of **2a**

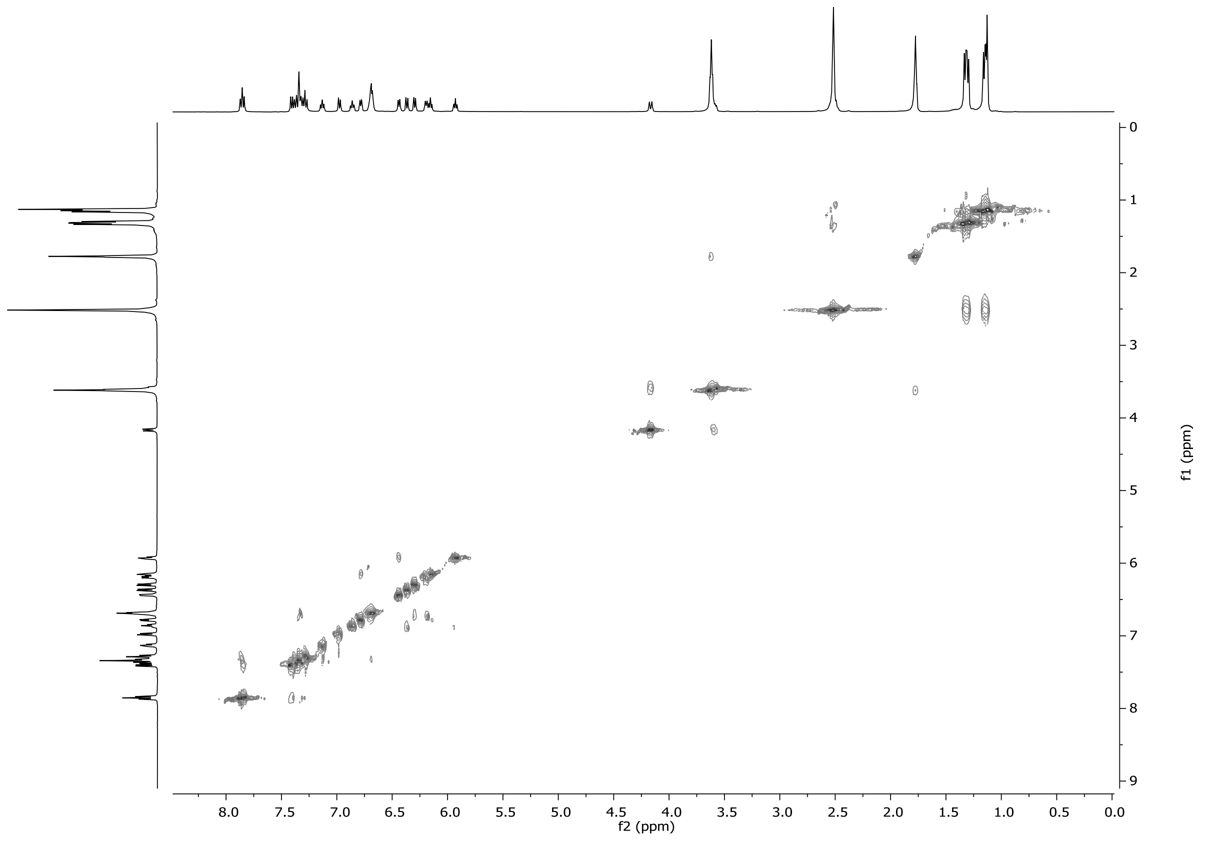

Figure S9-c: H-H COSY spectrum (DMSO-d_6) of **2a**

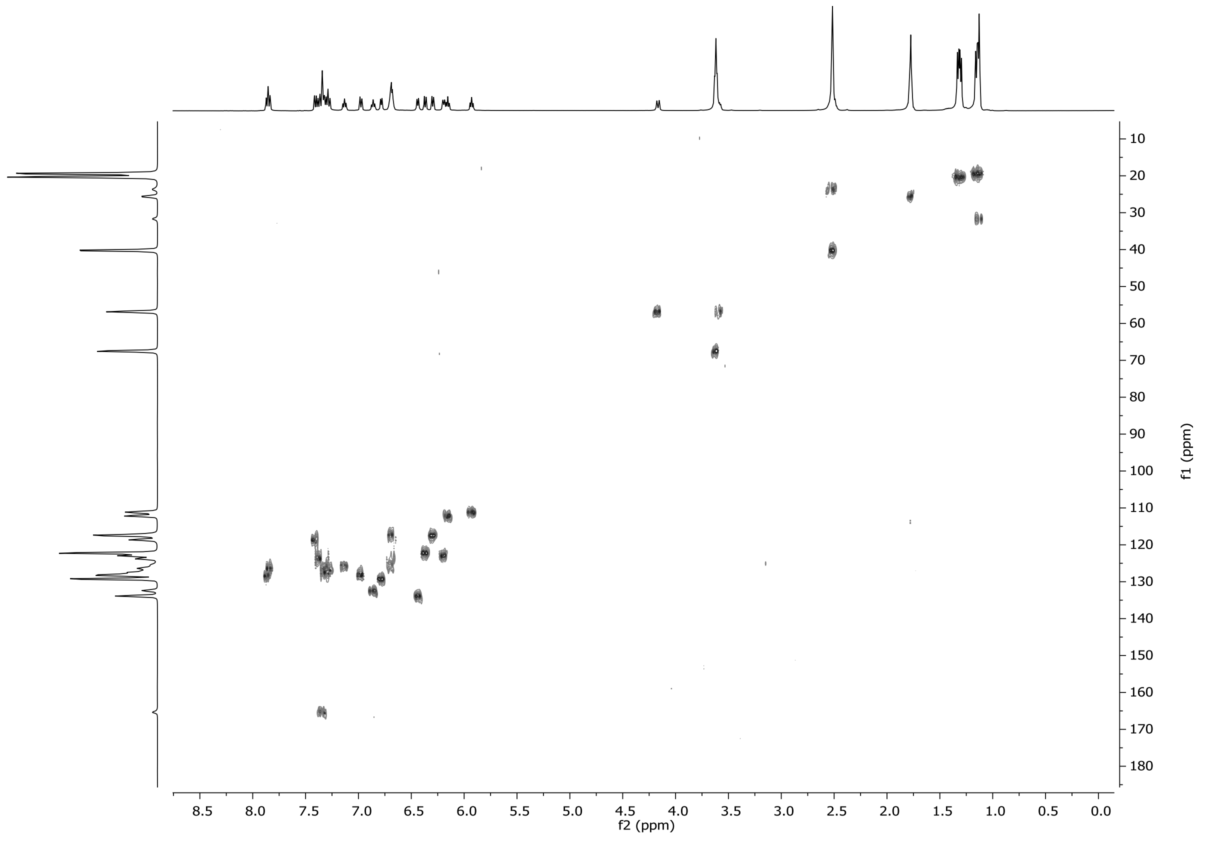

Figure S9-d: HSQC spectrum (DMSO-d_6) of **2a**

Ru(1-H$_2$)(^{13}CO)P(*i*-Pr$_3$)·MeCN (2-^{13}CO): 78.5 mg (0.16 mmol) of Ru(Cl)$_2$(DMSO)$_4$ was transferred to a flame dried Schlenk flask equipped with a reflux condenser and the vessel was purged three times with argon-vacuum cycles. Then 3 mL of ^{13}C-labelled methanol was added, followed by the addition of 0.11 mL (0.56 mmol) of P(*i*-Pr)$_3$. The colourless solution with suspended yellow solids was heated under reflux until the solid had dissolved and the solution turned orange-red (approximately 5 minutes). In the meantime 81.1 mg (0.16 mmol) of the (R)-2,2'-bis(salicylideneamino)-1,1'-binaphthyl (**1**) was transferred to a different flame dried Schlenk flask, which was purged three times with argon-vacuum cycles. To this yellow solid, 3 mL of ^{13}C-labelled methanol and 8 mg (0.35 mmol) of solid sodium where added. The resulting yellow suspension was added via a syringe to the stirring solution containing the ruthenium phosphine complex. The reaction mixture was heated for 55 min which resulted in a dark red solution that gave an orange-yellow precipitate upon concentration of the solvent to ±1 mL. After standing one day at 5 ˚C, the filtrate was taken off and the orange-yellow solid was dried and subsequently recrystallized from 2 mL hot acetonitrile, giving the orange-yellow crystalline complex in pure form. The acetonitrile filtrate was cooled in the freezer at -20 ˚C giving a second crop of **2**-^{13}CO. Overall yield: 65.7 mg, 51 %. **^{1}H** NMR (300 MHz, MeCN-*d*$_3$): δ 8.12 (d, *J* = 8.8 Hz, 1H), 8.02 (d, *J* = 8.2 Hz, 1H), 7.77 – 7.56 (m, 4H), 7.52 (t, *J* = 7.6 Hz, 1H), 7.44 – 7.28 (m, 2H), 7.29 – 7.13 (m, 2H), 7.13 – 6.87 (m, 4H), 6.76 (d, *J* = 8.6 Hz, 1H), 6.66 (d, *J* = 8.6 Hz, 1H), 6.55 (d, *J* = 8.0 Hz, 1H), 6.39 (t, *J* = 7.3 Hz, 1H), 6.20 (d, *J* = 7.9, 1.9 Hz, 1H), 6.06 (t, *J* = 7.3 Hz, 1H), 5.40 – 5.27 (m, 1H), 4.60 (t, *J* = 10.4 Hz, 1H), 3.80 – 3.60 (m, 1H), 2.76 – 2.39 (m, 3H), 1.38 (dd, *J* = 13.7, 7.2 Hz, 10H), 1.14 (dd, *J* = 12.5, 7.2 Hz, 9H). **^{31}P** NMR (122 MHz, Acetonitrile-*d*$_3$): δ 60.43 (d, *J* = 17.7 Hz). **^{13}C** NMR-CO (75 MHz, Acetonitrile-*d*$_3$): δ 205.30 (d, *J* = 16.9 Hz).

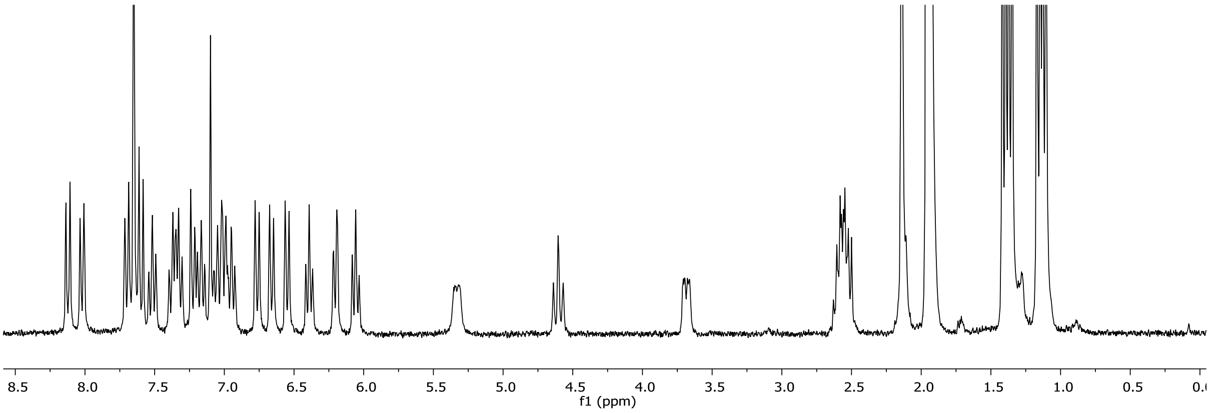

Figure S10: ^{1}H-NMR spectrum (MeCN-*d*$_3$) of **2-^{13}CO**

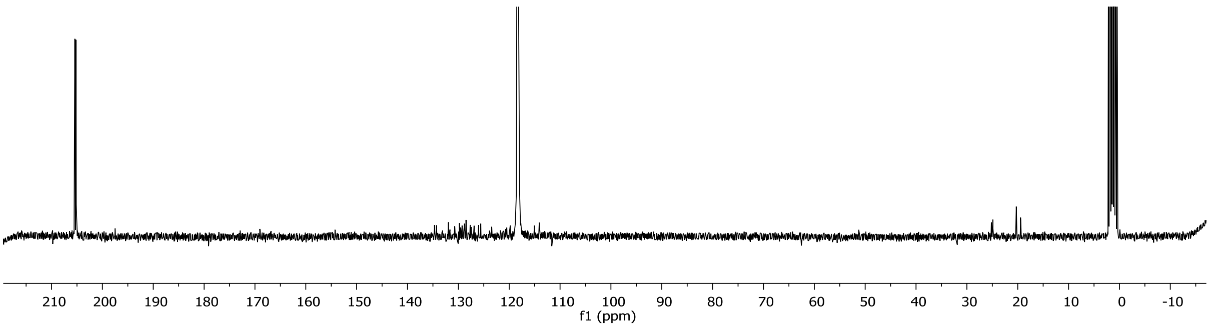

Figure S11: ^{13}C-NMR spectrum (MeCN-d_3) of **2-^{13}CO**

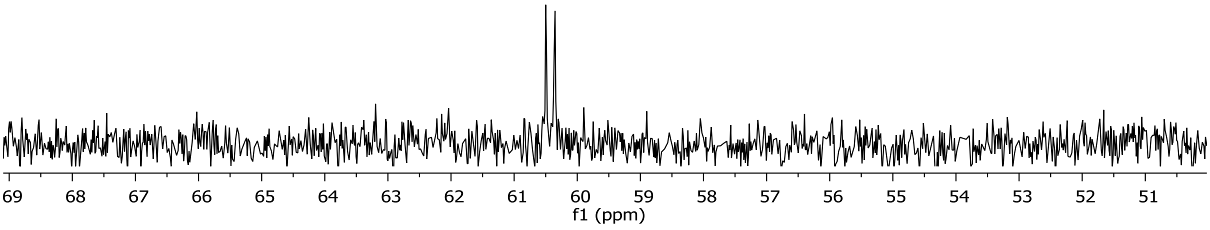

Figure S12: ^{31}P-NMR spectrum (MeCN-d_3) of **2-^{13}CO**

Complex **2**-D_2 was prepared using the same procedure as for **2**-^{13}CO, using CD_3OD instead of $^{13}CH_3OH$.

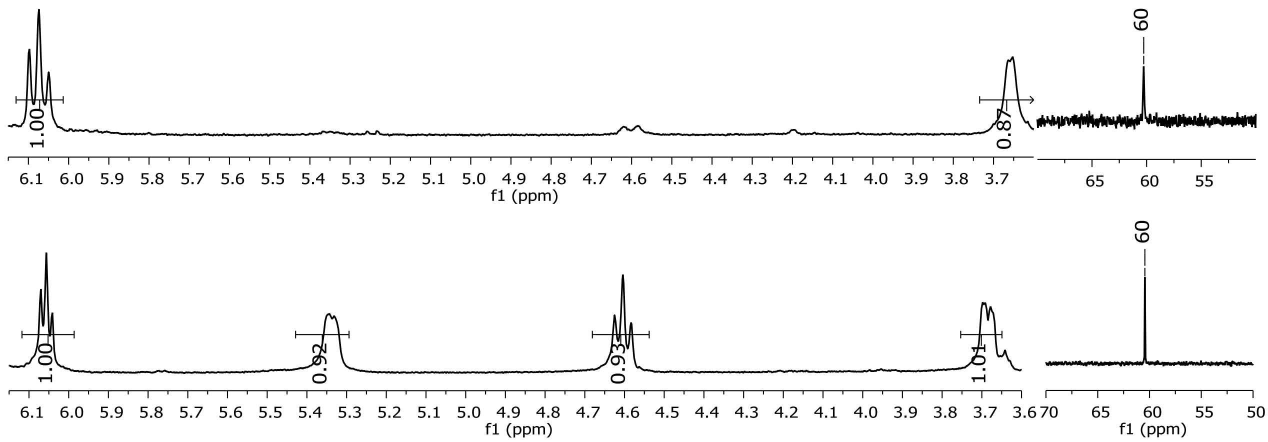

Figure S13: ^{1}H-NMR spectrum (left) and ^{31}P-NMR spectrum (right) (acetonitrile-d_3) of complex **2** (bottom) and complex **2-D$_2$** (top)

Ru(1)(DMSO)$_2$ (3):

103.4 mg (0.21 mmol) of (R)-2,2'-bis(salicylideneamino)-1,1'-binaphthyl (**1**) and 95.4 mg (3.98 mmol) of NaH were transferred to a Schlenk flask and purged 3x with vacuum argon cycles. Then 4.5 mL of THF was added and this suspension was stirred until gas evolution had ceased. The yellow solution

was filtered off, the white solid was washed with 3x 2mL THF and the combined filtrates were evaporated to dryness. To this yellow solid was added 64.9 mg (0.11 mmol) of [Ru(Cl)$_2$ *p*-cymene]$_2$ which was purged 3x with vacuum argon cycles. Then 10 mL of MeCN was added and the solution was stirred overnight. The resulting orange suspension was filtered and the solids were washed with DCM. The filtrates were combined and evaporated to dryness resulting in an orange red solid. This solid was dissolved in 3 mL DMSO and stirred overnight. After evaporation of the DMSO and *p*-cymene, the product was obtained in pure form. Yield: 159.3 mg, 99%

^{1}H NMR (300 MHz, Methanol-*d*$_4$) δ 8.47 (s, 1H), 8.02 (dd, *J* = 8.5, 4.7 Hz, 2H), 7.75 (dd, *J* = 8.4, 5.4 Hz, 2H), 7.64 (s, 1H), 7.61 (d, 1H), 7.48 (t, *J* = 8.1, 6.7, 1.4 Hz, 1H), 7.40 – 7.02 (m, 6H), 6.91 (d, *J* = 8.6 Hz, 1H), 6.76 (dd, *J* = 8.6, 4.5 Hz, 2H), 6.66 (d, *J* = 8.0, 1.8 Hz, 1H), 6.54 (t, *J* = 7.4 Hz, 1H), 6.27 (t, *J* = 7.4 Hz, 1H), 3.06 (s, 3H), 3.03 (s, 3H), 2.64 (s, 3H), 1.43 (s, 3H).

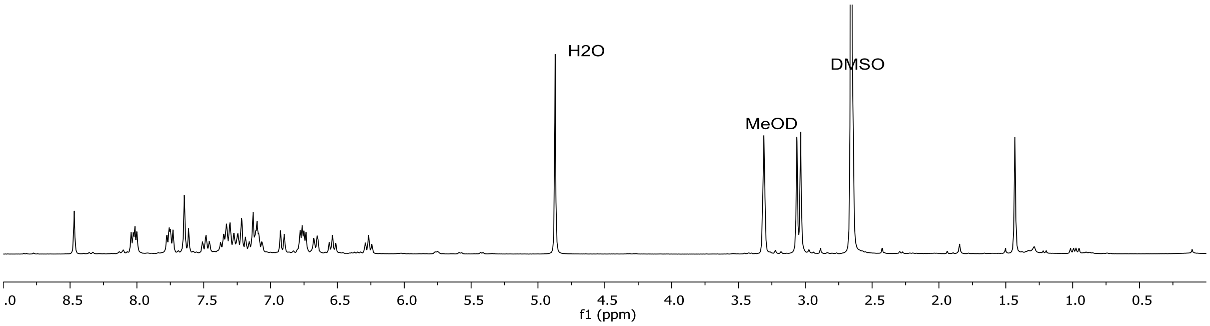

Figure S14: ^{1}H NMR spectrum (MeOD-*d*$_3$) of **3**

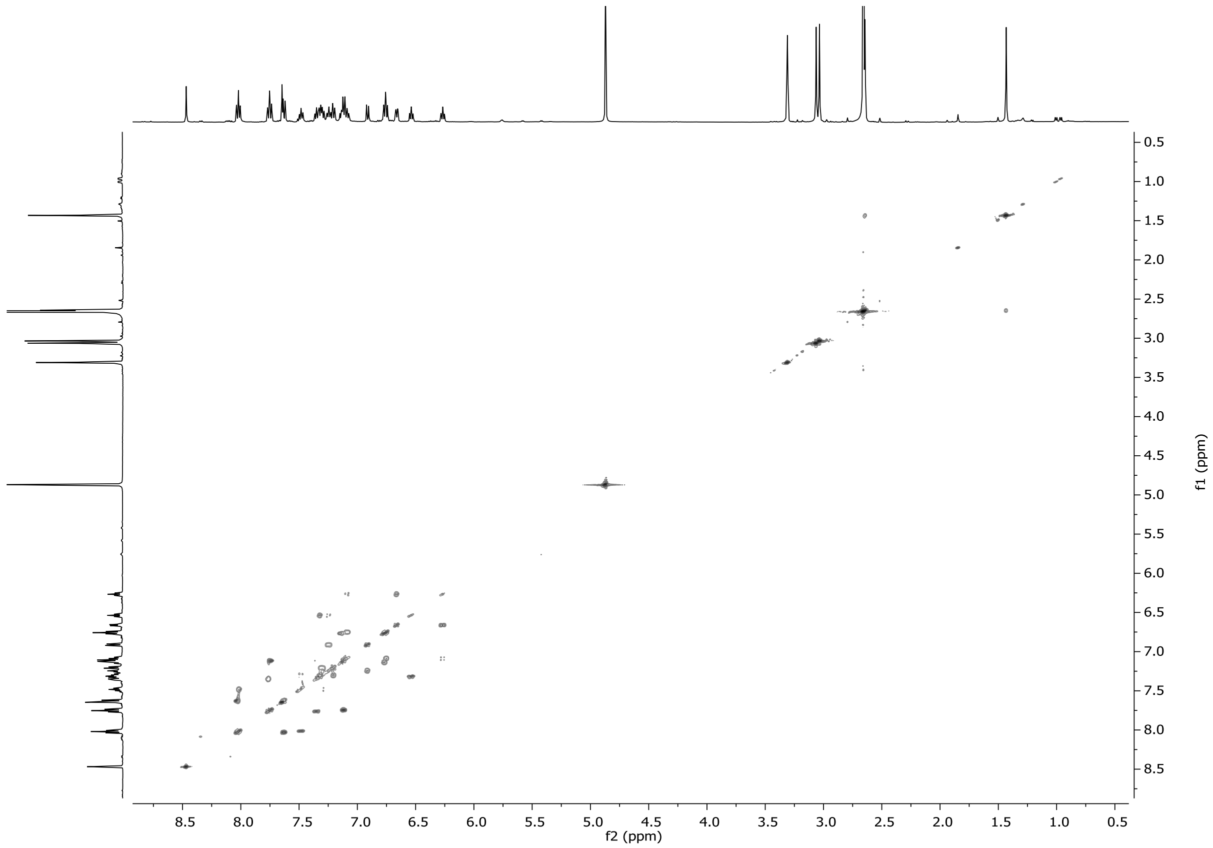

Figure S15: H-H COSY spectrum (MeOD-*d*$_3$) of **3**

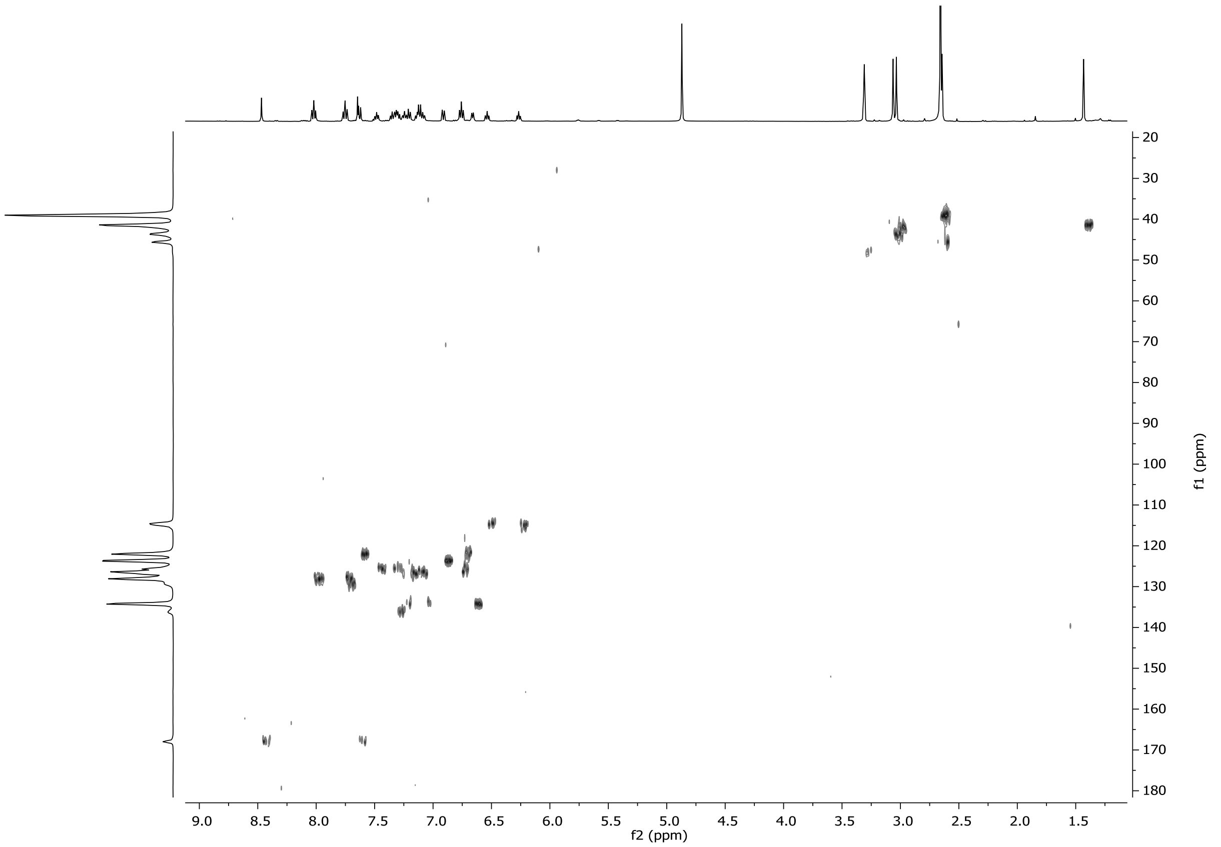

Figure S16: HSQC spectrum (MeOD-d_3) of **3**

Synthesis of $Ru(CO)_2(H)_2(P\text{-}iPr_3)_2$ (4)

102.5 mg (0.214 mmol) of $Ru(Cl)_2(DMSO)_4$ was added to a flame dried schlenk and purged 3x with vacuum argon cycles. Then 10 mL of methanol and 83 μL (0.427 mmol) of P-*i*Pr_3 were added and this was heated to reflux for 1h turning into a red solution. The solution was cooled to room temperature, 0.85 mL of LiOMe (1M, 0.85 mmol) was added and stirred for 5 minutes after which the solution was heated to reflux and stirred overnight yielding an orange/brown solution. The solution was filtered and evaporated to dryness in vacuo. The solid was extracted with dcm and the yellow product was crashed out adding pentane to this solution giving a yellow solid that matched literature data[4]. The solid was used without further purification for catalysis.

3) Hydrogen evolution experiments

Typical gas evolution experiment

The specified amount of base was transferred to a flame dried setup as depicted in Figure 21 and purged 3x with vacuum argon cycles until point "A". Then 29 mL of solvent (20.3 mL of methanol, 2.2

mL of water and 6.5 mL of dioxane) was added and the mixture was heated to reflux, while flushing the tube and the cylinder until point “B” with argon. When reflux temperature was reached the flushing was ended and heating was continued for 0.5-1h. Meanwhile, ~12-13 μmol of the specified ruthenium complex was transferred to another flame dried schlenk flask, purged 3x with vacuum argon cycles and subsequently dissolved in a total amount of 1.5 mL dioxane. This catalyst solution was added to the refluxing mixture and the measurement of gas evolution was started. The displacement of water in the cylinder (see S21 for the set up) was measured in time. All catalysis runs were measured in duplo and the gas formed was injected in the gas GC after every measurement. Molecular hydrogen was always the only gas present.

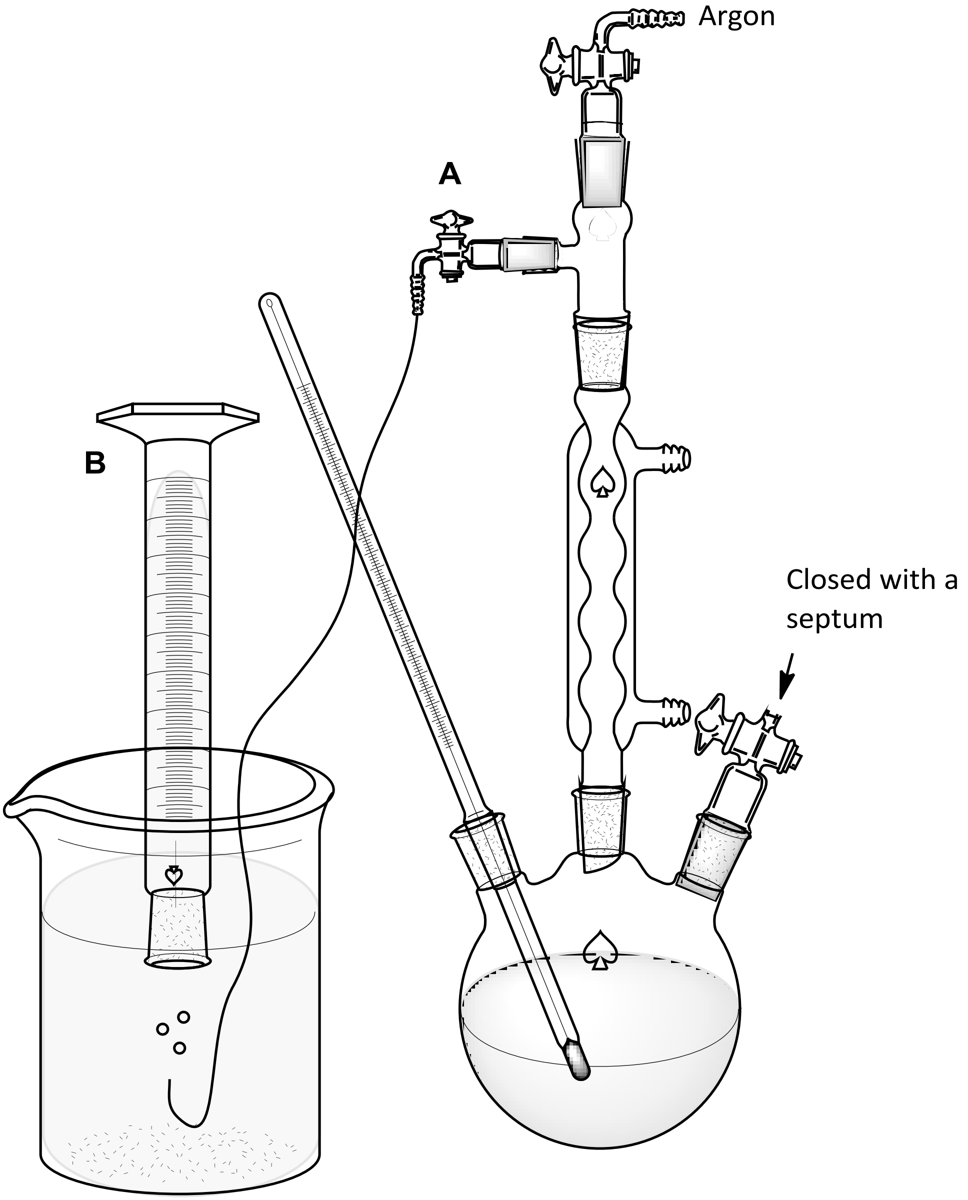

Figure S17: Set up for hydrogen evolution catalysis measurement

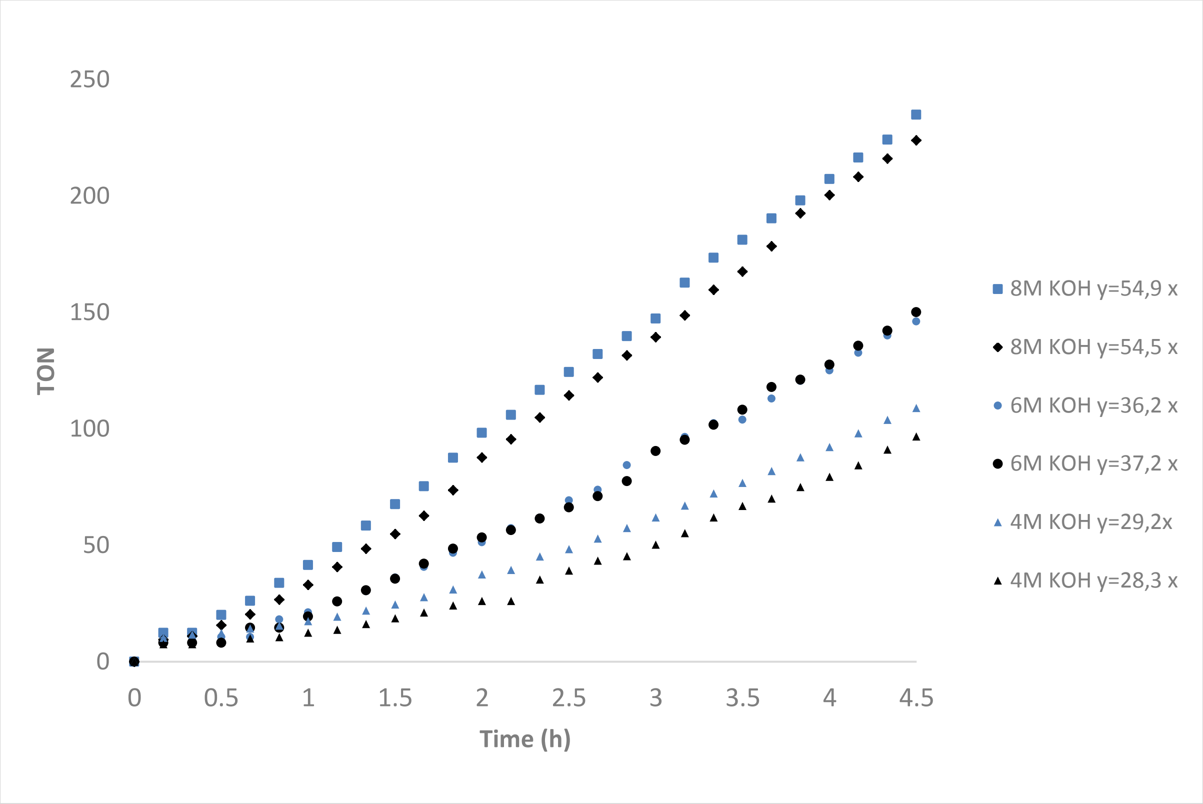

Figure S18: Hydrogen evolution with complex **2** as detected volumetrically using three different concentrations of KOH (8M KOH: squares; 6M KOH: dots, 4M KOH: triangles)

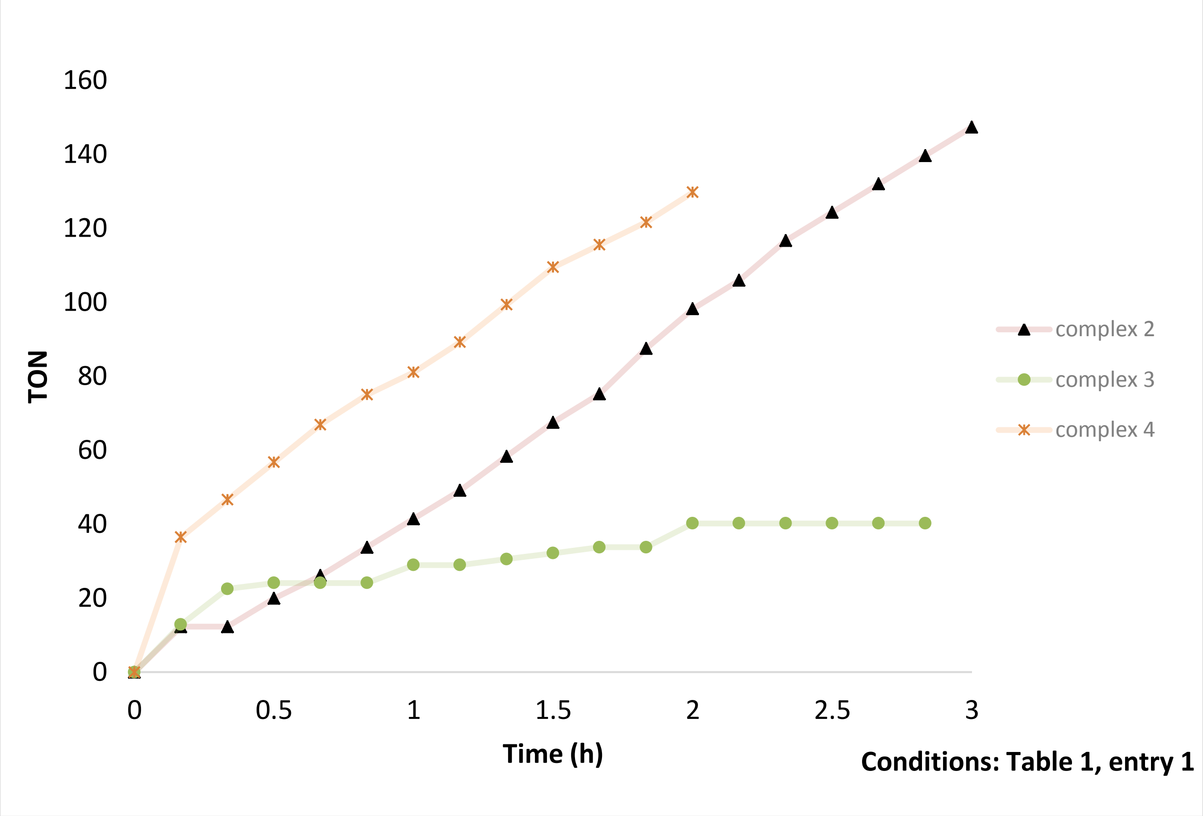

Figure S19: Hydrogen evolution using complex **2** (triangle), complex **3** (dot) and complex **4** (star) using conditions similar to 8M KOH in Figure S18.

TON is calculated using equation 1[5]

$$\mathrm{TON} = \frac{\mathrm{V_{obs}}}{\mathrm{V_m} * \mathrm{n_{cat}}} \qquad (1)$$

$\mathrm{V_{obs}}$: measured gas volume displacement cylinder [mL]

$\mathrm{V_m}$: molar gas volume: 24.49 [mL/mmol]

$\mathrm{n_{cat}}$: amount of catalyst [mmol]

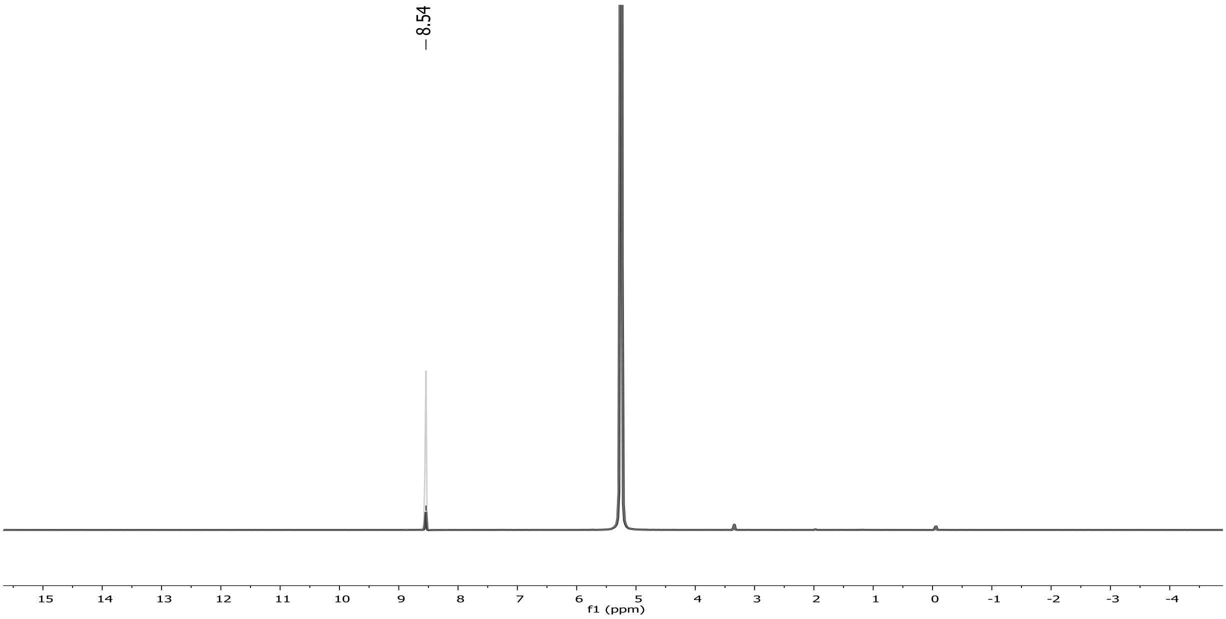

Figure S20: ^{1}H-NMR spectrum (D_2O) of solid after hydrogen production showing the formate (8.54 ppm) (black line); with additional formate added to the NMR tube (grey line).

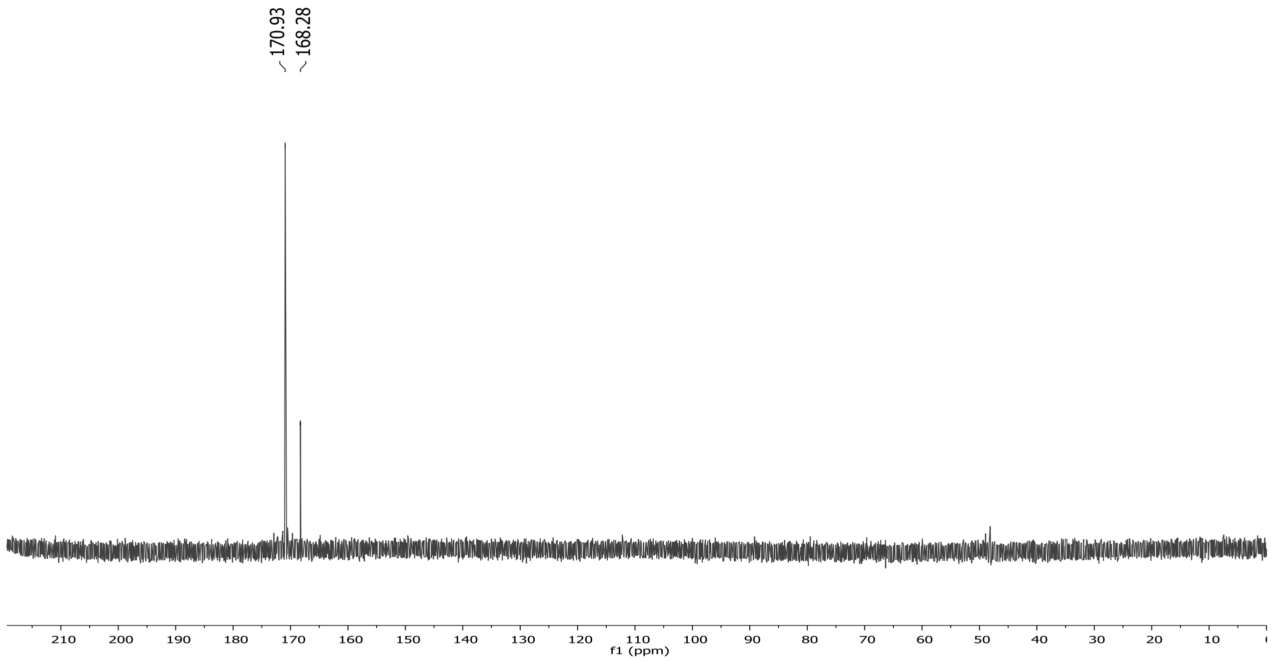

Figure S21: ^{13}C-NMR spectrum (D_2O) of solid after hydrogen production (Table 1, entry 1) showing the formate (170 ppm) and the carbonate (168 ppm)

4) Mechanistic investigations

Reaction of base with **2-^{13}CO**

Experiment 1: **2-^{13}CO**, methanol, water and KOH

11.1 mg (0.014 mmol) of **2-^{13}CO** was transferred to an oven dried HP NMR tube and purged 4x with vacuum argon cycles. 0.1 mL of a premade 8M KOH solution in methanol/water (9/1 v/v) followed by 0.4 mL of THF-d_4 was added. The solution was heated inside the NMR spectrometer at 100 ˚C, and the disappearance of the ^{13}CO peak was followed in time. A sample of the gas headspace was injected in the GasGC, showing only molecular hydrogen to be present. Dissolution of the precipitated white solid in D_2O showed presence of ^{13}C-labelled formate in both ^{1}H-NMR (8.25 ppm; the peak shift is effected by the amount of base present in solution) and ^{13}C-NMR (171.35 ppm).

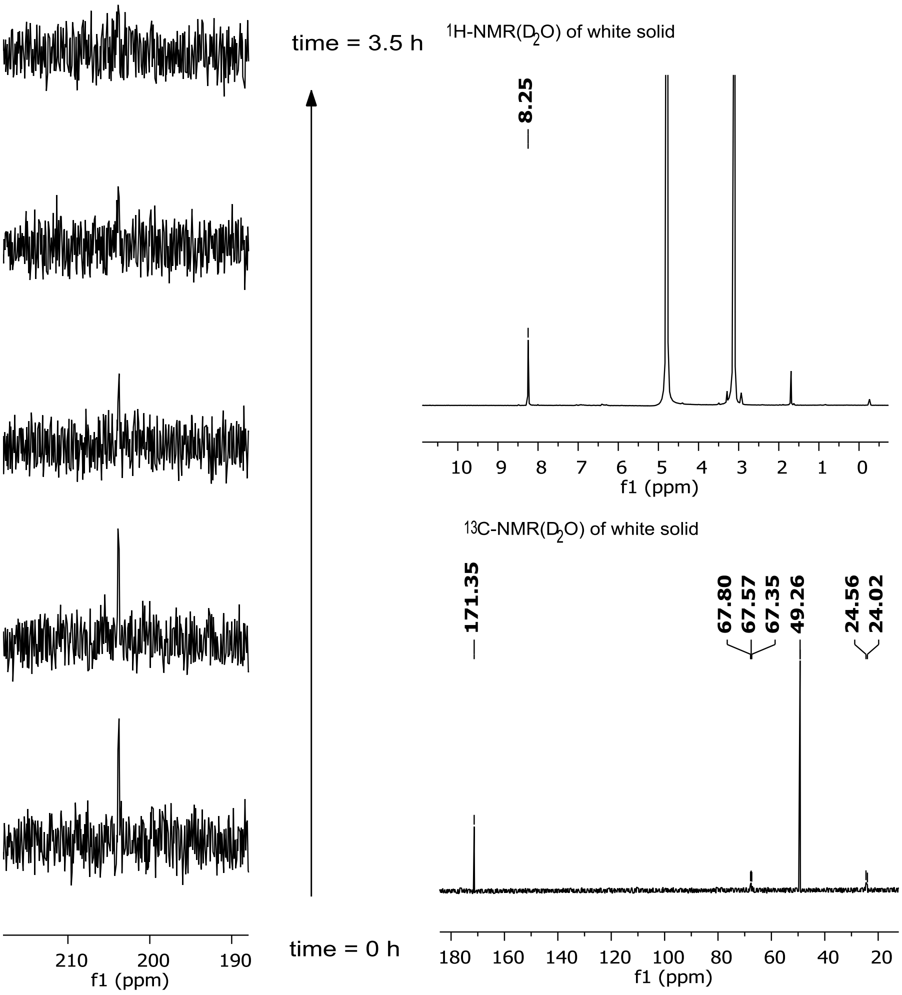

Figure S22: left: ^{13}C-NMR spectrum (THF-d_8) of experiment **1** in time tracking the disappearance of the ^{13}CO-ligand; right: ^{1}H-NMR and ^{13}C-NMR of white solid (D_2O) showing presence of only potassium formate (8.25 ppm and 171.35 ppm).

Experiment 2: Attempt to exchange **2-^{12}CO** for **2-^{13}CO** with $^{13}CD_3O^-$ under standard catalytic conditions

A typical gas evolution experiment was set up using 13.50 mg of **2**, in a 8M KOH solution of 8 mL dioxane, 9 mL CD_3OD, 11 mL $^{13}CD_3OD$ and 2.2 mL water. Catalysis was ran for 2h producing molecular hydrogen in a linear way (Figure 14). The reaction mixture was cooled to room temperature and extracted with 1 x 10 mL and 1 x 5 mL of dichloromethane. The organic solution was evaporated to dryness and dissolved in MeCN-*d_3*: no enriched ^{13}CO-signal could be detected in ^{13}C-NMR. ^{13}C-NMR of the methanol/water phase showed presence of the ^{13}C-enriched H/DCOO$^-$ indicating that the ^{13}C-labelled methanol was dehydrogenated. Thus, the carbonyl complex is not regenerated after the attack of base on the carbonyl.

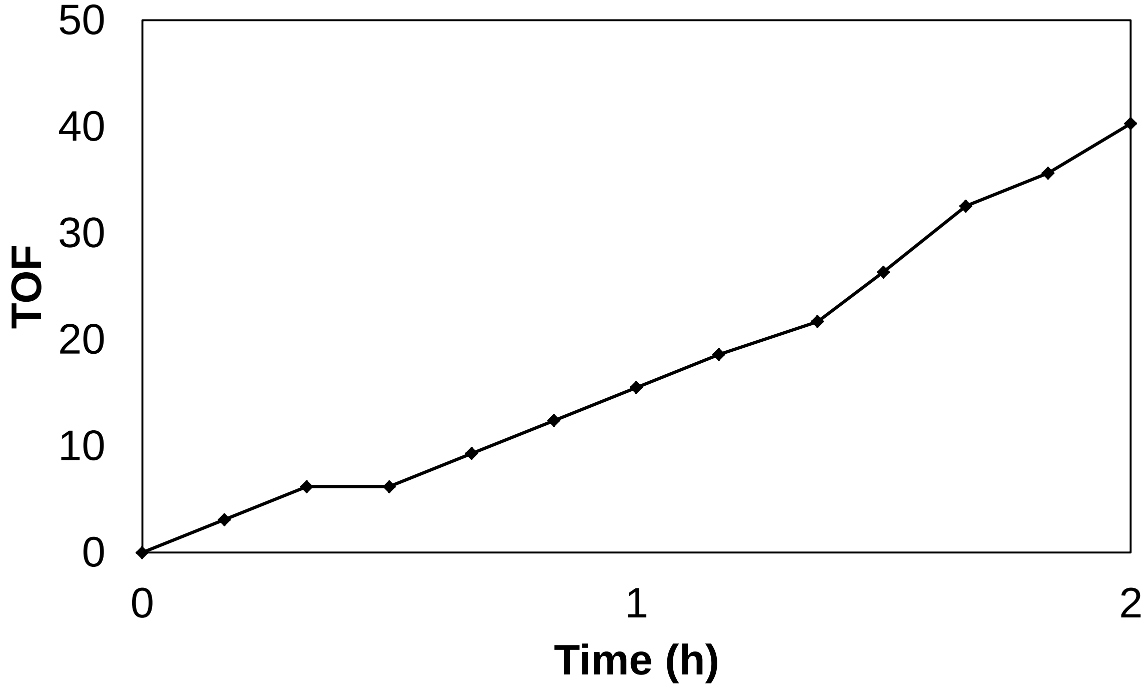

Figure S23: Hydrogen evolution from methanol while attempting to exchange **2-^{12}CO** for **2-^{13}CO** with $^{13}CD_3O^-$

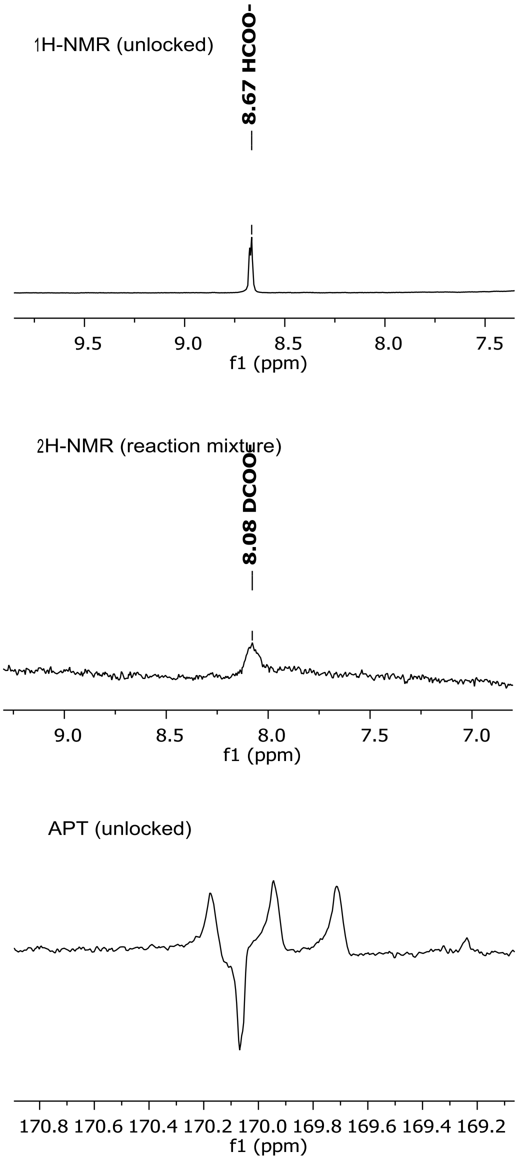

Figure 24: ^{1}H-, ^{2}H-NMR and APT of the reaction mixture.

5) Crystal data for **2**

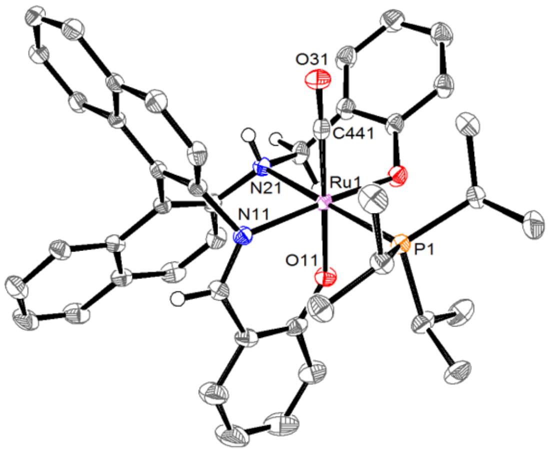

Figure 25: Ortep drawing of **2** (50 % probability ellipsoids). The second independent molecule, four acetonitrile molecules and most hydrogen atoms are omitted for clarity. Selected bond lengths [Å] and angles [°]: Ru1-P1 2.3431(9); Ru1-O11 2.069(2); Ru1-O21 2.088(2); Ru1-N11 2.063(3); Ru1-N21 2.285(3); Ru1-C441 1.825(4); Ru2-P2 2.3452(9); Ru2-O12 2.074(2); Ru2-O22 2.081(3); Ru2-N12 2.056(3); Ru2-N22 2.292(3) Ru2-C442 1.821(4); P1-Ru1-O11 92.35(8); P1-Ru1-O21 85.36(8); P1-Ru1-N11 100.95(9); P1-Ru1-N21 173.99(8); P1-Ru1-C441 91.30(11); O11-Ru1-O21 83.60(10); O11-Ru1-N11 88.14(10); O11-Ru1-N21 83.57(11); O11-Ru1-C441 175.23(14); O21-Ru1-N11 169.82(11); O21-Ru1-N21 89.79(10); O21-Ru1-C441 93.64(13); N11-Ru1-N21 83.37(11); N11-Ru1-C441 94.18(14); N21-Ru1-C441 92.55(14); N12-Ru2-C442 93.69(13); N22-Ru2-C442 92.79(14); O12-Ru2-O22 83.77(10); O12-Ru2-N12 88.06(11); P2-Ru2-O12 92.84(8); P2-Ru2-O22 84.95(8); P2-Ru2-N12 100.92(9); P2-Ru2-N22 173.64(8); P2-Ru2-C442 91.06(11); N12-Ru2-N22 83.89(11); O22-Ru2-N12 170.18(11); O12-Ru2-N22 83.10(11); O12-Ru2-C442 175.35(14); O22-Ru2-N22 89.72(11); O22-Ru2-C442 94.07(13).

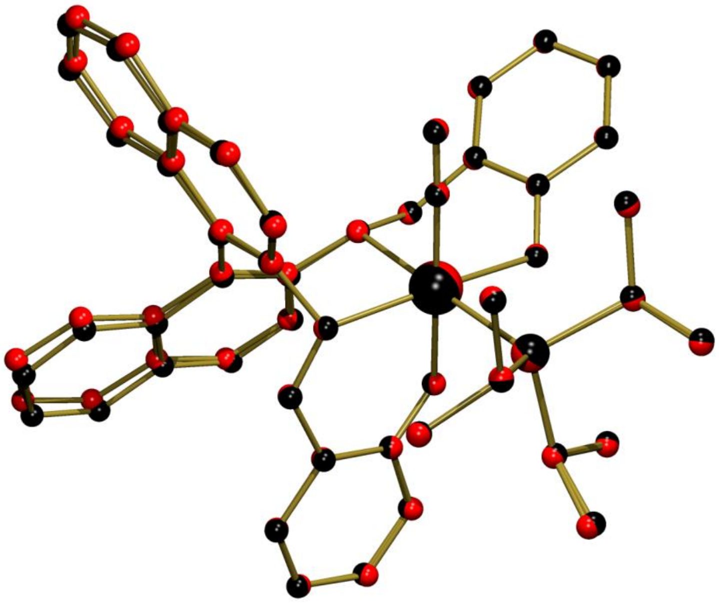

Figure 26: Quaternion fit of the two independent molecules in l0586a. The two molecules are approximately related by a non-crystallographic twofold rotation about uvw=[0,0,1]. This relation corresponds to the twin relation present in the crystal.

X-ray crystal structure determination of **2 · 2CH$_3$CN**

$C_{44}H_{45}N_2O_3PRu \cdot 2CH_3CN$, Fw = 863.96, yellow-orange block, 0.28 × 0.23 × 0.16 mm^3, triclinic, P1 (no. 1), a = 11.7197(5), b = 11.7886(5), c = 16.9812(9) Å, α = 103.014(2), β = 90.074(1), γ = 111.683(2)°, V = 2114.84(16) Å^3, Z = 2, D_x = 1.357 g/cm^3, μ = 0.46 mm^{-1}. The crystal appeared to consist of three fragments. The first and second fragment are related by a twin relation with a twofold rotation about *uvw*=[0,0,1] as twin operation. The third fragment has an arbitrary orientation of 3.9 ° with respect to the second fragment. Because of this crystal splitting, three orientation matrices were used for the integration with the Eval15 software[6]. 51792 Reflections were measured on a Bruker Kappa ApexII diffractometer with sealed tube and Triumph monochromator (λ = 0.71073 Å) at a temperature of 150(2) K up to a resolution of $(\sin \theta/\lambda)_{max}$ = 0.65 Å^{-1}. Multiscan absorption correction and scaling was performed with TWINABS[7] (correction range 0.72-0.75). 20002 Reflections were unique (R_{int} = 0.026), of which 19336 were observed [I>2σ(I)]. The structure was solved with Patterson overlay methods using SHELXT.[8] Least-squares refinement was performed with SHELXL-2013[9] against F^2 of all reflections using a HKLF-5 reflection file[10]. Non-hydrogen atoms were refined freely with anisotropic displacement parameters. N-H hydrogen atoms were located in difference Fourier maps and were refined freely with isotropic displacement parameters. All other hydrogen atoms were introduced in calculated positions and refined with a riding model. 1053 Parameters were refined with 3 restraints (floating origin). R1/wR2 [I > 2σ(I)]: 0.0225 / 0.0560. R1/wR2 [all refl.]: 0.0239 / 0.0566. S = 1.027. Batch scale factors of the second and third crystal fragment BASF = 0.3085(8)/0.1082(7). The Flack parameters[11] were determined by refining the structure against the non-overlapping reflections of the three fragments, resulting in x = -0.004(6), -0.020(9), and -0.012(16) for the three fragments, respectively. Residual electron density between -0.44 and 0.49 e/Å^3. Geometry calculations and checking for higher symmetry were performed with the PLATON program[12].

CCDC 1477274 contains the supplementary crystallographic data for this paper. These data can be obtained free of charge from The Cambridge Crystallographic Data Centre via www.ccdc.cam.ac.uk/data_request/cif.

References:

(1) Shen, Y.-M.; Duan, W.-L.; Shi, M. *J. Org. Chem.* **2003**, *68*, 1559.

(2) Alamsetti, S. K.; Sekar, G. *Chem. Commun.* **2010**, *46*, 7235.

(3) Brown, M. F.; Cook, B. R.; Sloan, T. E. *Inorg Chem* **1975**, *14*, 1273.

(4) Werner, H.; Esteruelas, M. A.; Meyer, U. *Chem. Ber.* **1987**, *120*, 11.

(5) Nielsen, M.; Alberico, E.; Baumann, W.; Drexler, H.-J.; Junge, H.; Gladiali, S.; Beller, M. *Nature* **2013**, *495*, 85.

(6) Schreurs, A. M. M.; Xian, X.; Kroon-Batenburg, L. M. J. *J. Appl. Crystallogr.* **2010**, *43*, 70.

(7) G. M. Sheldrick (2008). SADABS and TWINABS. Universität Göttingen, Germany.

(8) G.M. Sheldrick. Acta Cryst. (2015). A71, 3-8.

(9) G. M. Sheldrick. Acta Cryst. (2015). C71, 3-8.

(10) R. Herbst–Irmer, G. M. Sheldrick, Acta Cryst. 1998, B54, 443-449.

(11) S. Parsons, H.D. Flack, T. Wagner. Acta Cryst. (2013). B69, 249-259.

(12) A. L. Spek, Acta Cryst. (2009). D65, 148-155.
